# Virus distributions in wild bees are associated with floral communities at local to landscape scales

**DOI:** 10.1002/eap.70133

**Published:** 2025-11-11

**Authors:** Idan Kahnonitch, Katie F. Daughenbaugh, Na'ama Arkin, Tal Erez, Achik Dorchin, Michelle L. Flenniken, Nor Chejanovsky, Asaf Sadeh, Yael Mandelik

**Affiliations:** ^1^ Department of Entomology, Institute of Environmental Sciences, Faculty of Agriculture, Food, and Environment The Hebrew University of Jerusalem Rehovot Israel; ^2^ Department of Agroecology and Natural Resources, Newe Ya'ar Research Center Agricultural Research Organization–Volcani Institute Ramat Yishay Israel; ^3^ Department of Plant Sciences and Plant Pathology Montana State University Bozeman Montana USA; ^4^ The Mina & Everard Goodman Faculty of Life Sciences Bar Ilan University Ramat Gan Israel; ^5^ Department of Entomology and Nematology, Institute of Plant Protection Agricultural Research Organization ‐ Volcani Institute Rishon Letzion Israel; ^6^ The Steinhardt Museum of Natural History Tel Aviv University Tel Aviv Israel; ^7^ Present address: Laboratory of Zoology, Research Institute for Biosciences Department of Biology ‐ Invertebrates University of Mons | Royal Museum for Central Africa in Tervuren Mons | Tervuren Belgium

**Keywords:** *Andrena*, flower composition, flower diversity, interspecific virus transmission, landscape effects, Mediterranean agroecosystem, pollinators, wild bee health

## Abstract

Bees are focal pollinators, essential for maintaining biodiversity and crop production. Thus, reports of high annual honey bee colony losses and population declines among many wild bees in different parts of the world are of major concern. The spread of viruses is highlighted as a potential threat to bee communities. Viruses infect a wide range of bee species and can be transmitted interspecifically through shared floral resources. Therefore, the role of flowers as hubs of bee virus transmission requires a community ecology perspective. Here, we investigate local and landscape‐scale characteristics of floral communities potentially associated with the spread of viruses in the solitary *Andrena* spp. (mining bees). We surveyed 14 sites in a Mediterranean agroecosystem with varying local densities of honey bee (*Apis mellifera*) foragers and diversity of flowering species and assessed the prevalence of four common Hymenoptera‐associated viruses (deformed wing virus [DWV], black queen cell virus [BQCV], sacbrood virus [SBV], and Lake Sinai virus‐2 [LSV‐2]) in co‐foraging honey bees and mining bees. We found that the probability of virus presence in mining bees was generally associated with the diversity and composition of the local (site level) floral community, and with floral resource availability at the landscape scale (up to 1000‐m range). In addition, SBV and DWV prevalence in mining bees were positively related to the density of SBV‐infected, and total honey bee foragers, respectively. These findings demonstrate the focal role that the floral community at multiple spatial scales, and co‐foraging pollinator species, may play in virus spread and, potentially, pollinator health.

## INTRODUCTION

Pollinators, particularly bees, are central to the stability of both natural and agricultural ecosystems. Around 88% of angiosperm species rely on animal pollinators for reproduction (Ollerton et al., 2011), and 77% of the world's major food crops—especially edible fruits, nuts, and seeds—depend on pollination, mostly by bees (Klein et al., 2007). Alarmingly, global reports of wild bee declines and rising annual mortality in managed honey bee (*Apis mellifera*) colonies raise concerns for biodiversity conservation and food security (Aizen & Harder, 2009; Bartomeus et al., 2013; Bruckner et al., 2023; Carvalheiro et al., 2013; Garibaldi et al., 2022; Popovska Stojanov et al., 2021; Traynor et al., 2016; vanEngelsdorp & Meixner, 2010). Among the various drivers of pollinator declines, pathogens—particularly RNA viruses—are gaining attention for their potential impacts on both individual bee health and population dynamics (Genersch, 2010; Manley et al., 2015; Potts et al., 2010). Several RNA viruses originally studied in honey bees are now known to infect a broad range of bee species and other insect taxa, suggesting a wider host range than previously thought (Dobelmann et al., 2020; Grozinger & Flenniken, 2019; Tehel et al., 2016; Yañez et al., 2020).

In recent years, virus screening has expanded to include wild solitary bees such as those in the genus *Andrena* (mining bees)—a diverse and abundant group across temperate regions that plays a vital role in the pollination of both crops and wild plants (Michener, 1974; Schurr et al., 2019; Tang et al., 2019). Several viruses commonly found in honey bees have also been detected in *Andrena* species, including sacbrood virus (SBV; Dolezal et al., 2016; Murray et al., 2019; Ravoet et al., 2014), black queen cell virus (BQCV; Murray et al., 2019; Ravoet et al., 2014; Singh et al., 2010), Lake Sinai virus (LSV; Dolezal et al., 2016; Ravoet et al., 2014), and deformed wing virus (DWV; Dolezal et al., 2016; Murray et al., 2019; Radzevičiūtė et al., 2017; Singh et al., 2010). Moreover, *Andrena* bees have been shown to harbor actively replicating viruses, including DWV in *A. haemorrhoa* (Radzevičiūtė et al., 2017) and Andrena‐associated bee virus‐1 (AnBV‐1) in *Andrena* spp., which may be more closely linked to solitary bees than to honey bees (Daughenbaugh et al., 2021). Despite these findings, the epidemiological implications, fitness, and functional consequences of viral infections in solitary bees remain largely unknown (Kline et al., 2023). In light of the accumulated evidence for virus prevalence in *Andrena* and other non‐*Apis* bees, the potential risk that these viruses may pose is of concern. Therefore, understanding the ecological factors that shape virus spread in pollinator communities is critical for pollinator disease mitigation and conservation.

The mechanisms of virus transmission markedly differ between social and solitary bees due to major biological and behavioral differences. The highly eusocial honey bees live in dense colonies where behaviors like brood care, trophallaxis, and hygienic cannibalism facilitate rapid virus spread (Chen et al., 2006; Pfeiffer & Crowder, 2022; Posada‐Florez et al., 2021). Migratory beekeeping, global queen trade, and *Varroa destructor* mites—major vectors of several viruses—further contribute to interregional virus transmission (Nazzi et al., 2012; Wilfert et al., 2016). Phylogenetic and ecological studies have suggested that honey bees may act as competent hosts for interspecific virus transmission, contributing to the spillover of viruses to sympatric wild bees (Alger, Burnham, Boncristiani, & Brody, 2019; Fürst et al., 2014; Nanetti et al., 2021; Wilfert et al., 2016). However, patterns of virus prevalence and transmission dynamics are complex and likely vary by virus, host species, and ecological context (Daughenbaugh et al., 2021 for AnBV‐1, and McMahon et al., 2015 for ABPV).

One key route for interspecific transmission is through shared floral resources (Goulson & Hughes, 2015; McArt et al., 2014). However, the effectiveness of flower‐mediated virus transmission depends on flower traits (e.g., morphology, nectar accessibility), behavioral traits of visiting bees, and the specific pollinator‐pathogen‐flower combinations (Alger, Burnham, & Brody, 2019; Burnham et al., 2021; Mao et al., 2013; Spivak & Cariveau, 2020). Empirical studies have yielded mixed results, with some showing successful virus transfer via flowers and others failing to detect infection in sympatric foragers (Levenson & Tarpy, 2022; Singh et al., 2010; Yañez et al., 2015). Even if interspecific transmission is rare, it may have ecological relevance, especially in social species where within‐colony transmission can amplify viral prevalence.

For solitary bees like *Andrena*, virus spread likely relies on both flower‐mediated transmission and vertical transmission (Yañez et al., 2020). These routes may be sensitive to the degree of diet overlap among co‐foraging bee species. Many *Andrena* species are oligolectic, specializing in specific plant taxa, while generalists like honey bees exhibit broad foraging ranges (Cane & Sipes, 2006; Wcislo & Cane, 1996). Consequently, floral diversity and composition can influence pathogen dynamics by altering foraging overlap, exposure rates, and transmission efficiency. Community‐level plant–pollinator interactions and asymmetric flower‐sharing patterns further complicate these dynamics (Proesmans et al., 2021; Ruiz‐González et al., 2012).

While most studies focus on local factors, landscape‐scale processes, which shape pollinator foraging behavior and community structure, can indirectly influence patterns of viral prevalence (Kremen et al., 2007; McNeil et al., 2020). Landscape features, including floral resource availability (FRA) and abundance, shape viral prevalence by influencing pollinator movement patterns and interactions among co‐occurring species (Piot et al., 2020; Proesmans et al., 2021). Landscape simplification, for example, has been linked to reduced pathogen prevalence in *Bombus* bees, likely due to altered dietary breadth and plant–pollinator interaction networks (Figueroa et al., 2020). In addition, pollinators that can fly long distances (like honey bees) may spread viruses at considerable landscape scales in their foraging bouts. This is further amplified in fragmented landscapes or areas with depleted floral resources, where pollinators may be forced to travel greater distances, thereby increasing potential interspecific contact and opportunities for virus transmission (Kremen et al., 2007). Yet the landscape‐scale determinants of viral infections in solitary bees remain understudied.

We conducted a field survey to investigate the factors and spatial scales that shape virus prevalence in *Andrena*. Specifically, we studied whether virus prevalence in *Andrena* is associated with (i) the diversity and composition of floral species at the site, (ii) land use and flower availability in the surrounding landscape, and (iii) the density of co‐foraging *Apis mellifera* at the site.

## METHODS

### Study region and bee fauna

We conducted a field survey during spring 2018 in the Judean Foothills, a unique ecosystem in central Israel, at the interface between the humid Mediterranean and the semiarid climatic zones. The landscape in this region consists of a mosaic of agricultural land, planted forests, Mediterranean shrubland and grassland, and developed areas (mainly rural settlements and roads). Although threatened by increasing development pressure, this ecosystem is considered a biodiversity hotspot (Sorek and Shapira, 2018) and sustains a diverse and abundant wild bee community (Pisanty et al., 2014, 2016; Pisanty & Mandelik, 2015). Commercial honey bees are prevalent in this region and managed for crop pollination and honey production; feral honey bees are considered absent in this region, likely due to *Varroa* mites (Soroker et al., 2018) and possibly other factors. Prior to fieldwork, we conducted a systematic survey of the study area, recorded the locations of apiaries (Appendix S1: Figure S1), and contacted their owners to verify that the hives were stationary and had not been relocated since the preceding fall. A preliminary field survey in the study region indicated that wild bees of the genus *Andrena* (Andrenidae) are the most dominant bees in spring. In the study region, 45 and 41 *Andrena* species were recorded in the current study and in a previous one (Roth et al., 2023), respectively. However, the actual local *Andrena* species richness is likely higher.

### Survey design

**FIGURE 1:**
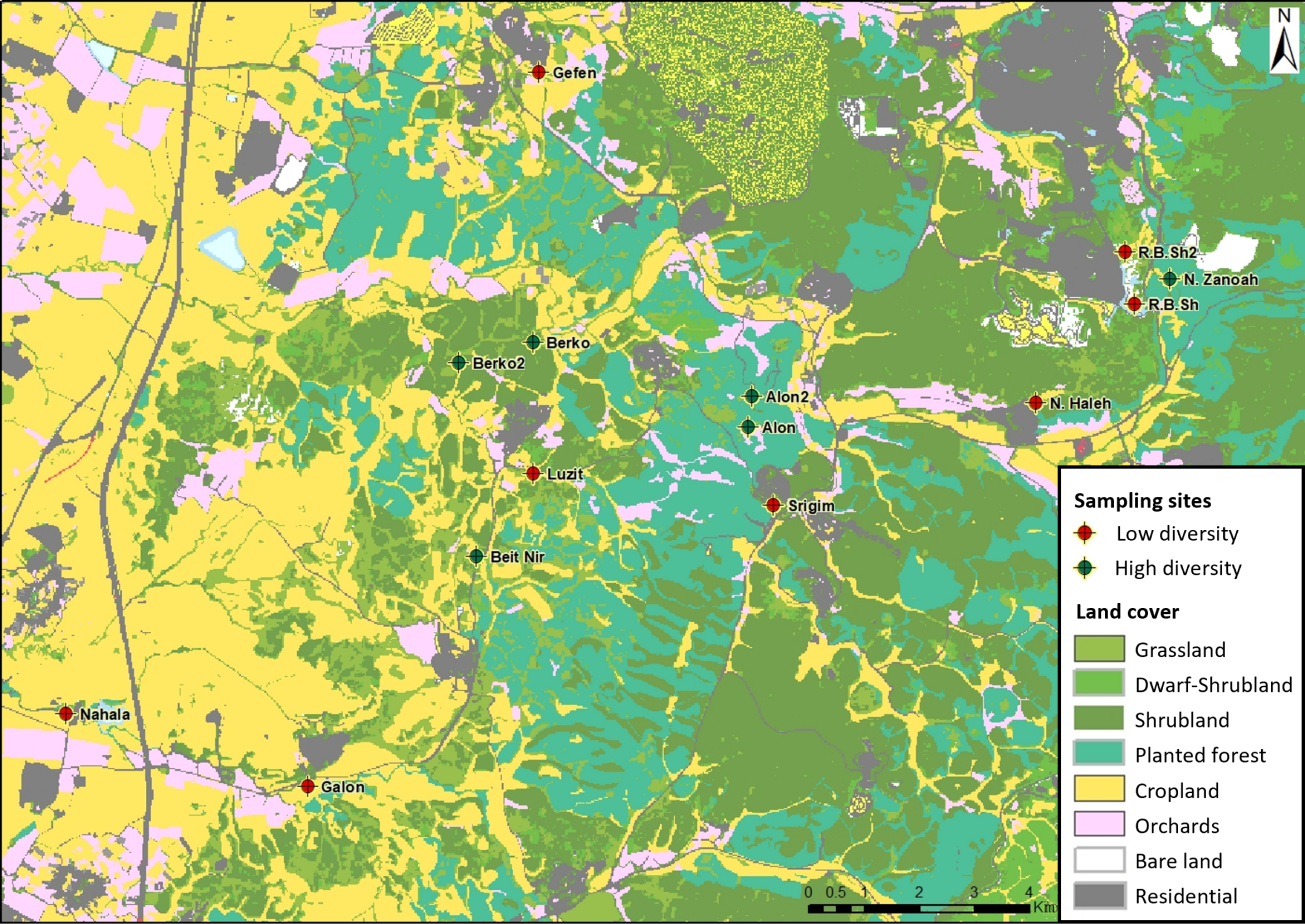
Map of the study area (Judea foothills, central Israel) with sampling sites (*N* = 14) labeled and marked with green and red dots indicating high and low floral diversity, respectively, and surrounding land‐cover.

The survey included a total of 14 sites. Each site comprised a 625‐m^2^ plot located at the center of a larger wild bloom patch (mostly >3000 m^2^), dominated by yellow‐flowering Brassicaceae species, mainly *Sinapis alba*, *Hirschfeldia incana*, and *Rapistrum rugosum*. Sites were selected according to their flowering species richness (range: 1–14), to create a balanced representation of both high and low floral richness: eight sites had a relatively low floral richness (≤6 blooming floral species; median = 3), and the other six sites had a relatively high floral richness (≥9 blooming floral species; median = 11.5) (Figure 1). Plant species that were not visited by bees were excluded because their contribution to flower‐mediated virus spread within the studied bee communities is likely negligible. The average distance between neighboring plots was 1914 m, ranging between 524 and 4960 m.

### Landscape classification

To account for possible landscape effects on virus distribution, we first classified dominant land‐cover types in the surrounding landscape at 1‐km radii around each site. Using satellite images obtained from Israel's governmental mapping company (Survey of Israel; Govmap 2018), combined with ground truthing, we classified the following dominant land‐cover categories in our study region, namely, agricultural land (orchards, cropland), natural and seminatural habitats (shrubland, dwarf‐shrubland (phrygana), grassland, planted forest), developed and disturbed land (residential areas, fallows, road margins), and bare land (e.g., roads, paved area). Then, based on these land‐cover types, we calculated two landscape variables, within 250‐, 500‐, and 1000‐m radii around each site:

1. Proportion of non‐agricultural land. This was calculated based on the compilation of all land‐cover categories with wild bloom, including all natural and seminatural habitats and developed and disturbed land (excluding residential areas which have limited/no bloom). This variable is commonly used in landscape‐scale pollinator studies (e.g., Figueroa et al., 2020; Zou et al., 2017) as a proxy for pollinator habitat quality. The proportion of non‐agricultural land surrounding our study sites ranged between 27% and 100%, 23% and 99%, and 13% and 92% within 250, 500, and 1000 m, from study site centroids, respectively.
2. FRA. The FRA is a pollinator resource‐based approach developed by Lonsdorf et al. (2009) to model pollinator activity and implemented by Lotan et al. (2018) to estimate the value of different land‐cover types to Israel's pollinators. The FRA index is based on assigning to each land‐cover type an expert‐based relative value reflecting its typical floral resource density during the relevant blooming period (i.e., February through March in the study area). Specifically, for each land‐cover type, the index is calculated as the floral density factor (ranging from 0 to 1) multiplied by the proportion of the blooming period during which it is actually in bloom (ranging from 0 to 1). Finally, the FRA score is calculated as the weighted average value of the different land‐cover types around each site. FRA scores varied considerably among sites (0.20–0.71, 0.20–0.50, and 0.22–0.40 within 250, 500, and 1000 m from study site centroids, respectively).

### Field sampling and floral community metrics

Field work was conducted in mid‐March, corresponding to peak spring bloom, and lasted 2 weeks, to minimize temporal variation in plant and bee community compositions between sites. Each site was sampled once between 8:00 and 16:00 when weather conditions were favorable for bee activity (temperature >17°C, wind velocity <3 m/s, and clear or partially clear skies). Foraging bee sampling was carried out for a total of 40 person‐minutes (excluding handling time of captured bees) and conducted by slowly walking throughout the plot and hand‐netting any bee that was observed landing on a flower, while keeping records of the visited flower species. Each captured bee was identified in situ to the lowest taxonomic level possible, immediately placed in a vial, and kept on dry ice. We collected additional foraging bees as needed to attain a sample of at least 11 *Andrena* and 11 *A. mellifera* individuals per site for viral screening. At the end of each sampling day, all the collected specimens were brought to the lab and stored at −80°C. The sample of mining bees that was used for viral screening consisted of the three most dominant *Andrena* species (collectively comprising >70% of the observed *Andrena* individuals in the study): *Andrena aerinifrons levantina* Hedicke (1938); *Andrena urfanella* Scheuchl and Hazir (2012); *Andrena ochraceohirta* Alfken (1935) (recently resurrected; Pisanty et al., 2022); and a closely related morphospecies from the subgenus *Truncandrena*. These four *Andrena* species foraged almost exclusively on yellow‐flowering mustard plants; collectively ~99% of their observed floral visits were recorded *on Sinapis alba, Hirschfeldia incana*, and *Rapistrum rugosum*, including in the high‐floral‐richness sites, where the relative abundance of these flower species was <67% of total site flower abundance. We considered these four *Andrena* species as a single epidemiological population owing to their high phylogenetic relatedness and similar diet, phenology, diurnal activity patterns, foraging behavior, and nesting habits.

In each site, we sampled the floral community using 10 1‐m radius hoops, placed randomly throughout the site and recorded the total number of floral units of each flowering species. For most of the species, the floral unit was defined as an individual flower or a distinct floret within sparse inflorescences. For Compositae, Umbelliferae, and *Trifolium*, which are characterized by dense inflorescences, the floral unit was defined as an individual inflorescence, but to account for variability in inflorescence size, we used a standard of ~1‐cm radii inflorescence and calculated the floral unit counts accordingly. We used this approach to account for bee tendency to visit many adjacent florets per visit on densely packed inflorescences, but only a few florets per visit on sparse inflorescences (IK, personal observations); this difference in visitation behavior possibly affects virus deposition on densely versus sparsely packed inflorescences. In this way, a floral unit will best represent a single bee visit, regardless of flower/inflorescence shape.

Floral diversity was calculated using the Shannon–Weaver index. To investigate how floral diversity may be linked to the potential overlap in floral resources between the focal *Andrena* spp. and the other pollinators in the site, we also examined the relationship between floral diversity and visitation density (by all bee taxa) on the yellow Brassicaceae floral plants using negative binomial generalized linear model (GLM) (glm.nb function from package MASS in R; Ripley et al., 2013). Yellow Brassicaceae floral abundance was included as an offset term, and the Wald Z statistic was used to test the model's coefficient. Similarity of floral composition among the sites was calculated using the first two axes of a correspondence analysis (CA; vegan package; Oksanen et al., 2020), in which we combined three *Trifolium* species into a single genus‐level group, and the three dominant yellow mustard species (*Sinapis alba*, *Hirschfeldia incana*, and *Rapistrum rugosum*) that were utilized non‐selectively by our focal *Andrena* species, into a single functional group. Flower species scores on the first two CA ordination axes were computed and used to characterize the relative association of specific flower species to each of the CA axes.

### Virus detection

From each site, virus identification efforts were focused on individual *A. mellifera* and *Andrena* (median = 11). High throughput sequencing identified regional virus genome sequence variations for BQCV and LSV‐2, which required the utilization of new primer sequences (Daughenbaugh et al., 2021). The presence and abundance of four common hymenopteran viruses, namely, BQCV, DWV, SBV, and LSV‐2, were assessed using the procedure of RNA extraction followed by cDNA preparation, polymerase chain reaction (PCR), and qPCR, as described in Daughenbaugh et al. (2021). The primers used in this analysis are detailed in “T1. Primers.xlsx” within the archived dataset (Kahnonitch et al., 2025). The analyses described herein utilized only presence/absence data in mining bee and honey bee populations; data on viral prevalence and abundance (qPCR) are provided in “T3. qPCR‐ Andrena sample.csv” and “T4. qPCR‐Apis mellifera sample.csv” within the archived dataset (Kahnonitch et al., 2025). To determine whether the virus prevalence for each of the four viruses differed significantly among the *Andrena* species that comprised our sample, we employed generalized mixed‐effects linear models (using the glmer function from the lme4 package in R; Bates et al., 2020), with binomially distributed errors and a logit link function and included random intercepts by sampling site. No significant differences were found in virus prevalence among the studied *Andrena* species (Likelihood Ratio Test; *p* > 0.1), thus further supporting our decision to combine these species into a single epidemiological group.

To evaluate the probability that viruses are shared among *A. mellifera* and *Andrena* spp., we compared the phylogenetic similarity of viral sequences. Nucleotide identity of more than 90% in viral sequences indicates virus sharing, as this level of variation is usually generated when viral genomes replicate (Lauring & Andino, 2010). We evaluated sequence similarity only for viruses that were likely shared according to virus prevalence data.

### Data analysis

We analyzed the presence/absence data of each of the four viruses in mining bees as a binary response variable, using generalized mixed‐effects linear models (glmer function from package lme4 in R; Bates et al., 2020) with binomially distributed errors and a logit link function, and with random intercepts by sampling site. We explored three groups of potential predictors: (1) The local flower community at each site, represented by two variables: floral diversity (Shannon–Weaver index) and floral composition (CA axes); (2) The surrounding landscape within 250, 500, and 1000 m around each site. As the landscape‐level predictor, we used the proportion of non‐agricultural land and the FRA index in separate analyses, to find whether a land‐use or a resource‐based perspective provides an overall better fit to the data, while reducing the number of models considered; (3) The density of co‐foraging *A. mellifera*, estimated from the 40‐min bee sampling. We examined both the density of all co‐foraging *A. mellifera* and the density of virus‐positive *A. mellifera* only (separately for each of the viruses). Virus‐positive *A. mellifera* densities were estimated by multiplying total *A. mellifera* density by the *A. mellifera* virus prevalence estimate in each site. To enable convergence, this variable was rescaled using division by 10 (Babyak, 2009).

Given the high proportion of negative detections in the *Andrena* sample, we considered the possibility of zero inflation in the data, which could indicate the need for an alternative modeling approach. To assess this, we conducted two diagnostic analyses: (1) a Zero Inflation Test (using the testZeroInflation function; DHARMa package; Hartig & Lohse, 2022), and (2) a model comparison, where we fitted a zero‐inflated model and compared its corrected Akaike information criterion (AIC_c_) score to that of our standard model (using the glmmTMB function; glmmTMB package; Brooks et al., 2017). For all viruses, these tests indicated no evidence of zero inflation in the data (Appendix S2).

Model selection was carried out using AIC_c_ (Akaike, 1987) and included all models within two units from the top model. Models within two AIC_c_ units of the null model (containing only the random intercept effect of site) were excluded. Multicollinearity was diagnosed using the function “vif” from the package “car” (Fox et al., 2012) with a cutoff value of 5. The variance inflation factor of the top model for DWV prevalence in mining bees was 8. Despite the mild multicollinearity in this model, the stability of coefficient signs was confirmed using perturbation analysis (after Belsley, 1991). The statistical significance of predictors in all selected models was determined using likelihood ratio tests. Statistical analyses were conducted in R version 4.2.1 (R Core Team, 2022).

## RESULTS

We screened 150 honey bee and 153 mining bee samples for the presence of several bee viruses and detected four honey bee‐associated viruses: BQCV, DWV, SBV, and LSV‐2. Virus prevalence in honey bees was very similar to the national averages (for BQCV, DWV, and SBV; Soroker et al., 2018) and, overall, was much higher than virus prevalence in mining bees. BQCV was detected in 8 mining bees from 4 sites and in 134 honey bees from all 14 sites, with mean prevalence of 5.2% (range: 0–27.3) and 83.76% (range: 9.1–100) per site, respectively; DWV was detected in 10 mining bees from 3 sites and in 76 honey bees from 11 sites, with mean prevalence of 6.36% (range: 0–45.5) and 51.08% (range: 0–100) per site, respectively; SBV was detected in 8 mining bees from 3 sites and in 31 honey bees from 7 sites, with mean prevalence of 5.2% (range: 0–54.6) and 20.08% (range: 0–100) per site, respectively; LSV‐2 was detected in 18 mining bees from 6 sites and in 125 honey bees from all 14 sites, with mean prevalence of 11.76% (range: 0–45.5) and 85.2% (range: 60–100) per site, respectively. These data are provided in “T2. Sites.xlsx,” within the archived dataset (Kahnonitch et al., 2025). For all four viruses, any site that had virus‐positive mining bees also had virus‐positive honey bees, but not vice versa. For both mining bees and honey bees, qPCR data indicated that some of these bees likely had active infections, as indicated by high viral abundance (Daughenbaugh et al., 2015; De Miranda et al., 2013). These data are provided in “T3. qPCR‐ Andrena sample.csv” and “T4. qPCR‐ Apis mellifera sample.csv” within the archived dataset (Kahnonitch et al., 2025).

We recorded 83,142 floral units belonging to at least 76 species of flower plants, out of which 30 species were seen visited by bees of any species during the field survey. While the focal *Andrena* species foraged almost exclusively on the three Brassicaceae yellow‐flowering plants, honey bees foraged on an additional 12 flower species (Appendix S3: Figure S1).

The models that used the proportion of non‐agricultural land as the landscape‐level predictor yielded, in most cases, worse fits than those using FRA score (Appendix S4: Table S1), and in some cases, multiple selected models for the same virus resulted in inconsistent interpretations (SBV in mining bees; Appendix S4: Table S2). Using the FRA score in the models resulted in overall better fits and more consistent interpretations, and we therefore consider it a superior landscape‐level predictor for our data. In Appendix S4, we also present the results of the models with the proportion of non‐agricultural land. The full details of all models are provided in Appendix S5.

### Virus prevalence in mining bees

Two similar models were selected for the probability of BQCV presence in mining bees, with predictors including the FRA score within 1000 m of the site, and floral composition (2nd CA axis) in the site. FRA within 1000 m was positively associated with BQCV prevalence in mining bees. However, the AIC_c_ score of this model was only moderately lower than that of the null model (ΔAIC_c_ = 2.32; Table 1).

**TABLE 1:** Selected models (within 2 AICc units of top model and >2 AICc units from the null model) of virus presence in ***Andrena***, using FRA score as the landscape‐scale predictor.

	Local floral community		Surrounding landscape (FRA score)	*A. mellifera* density
	Flower species diversity	Floral genus composition		
**BQCV**		(CA axis 2)	(1000 m range)	
Model 1	‐‐‐‐	2.02*	28.10*	‐‐‐‐
Model 2	‐‐‐‐	2.20*	40.40**	n.s.
**DWV**				(total)
Model 1	8.65**	‐‐‐‐	‐‐‐‐	3.25**
**LSV‐2**		(CA axis 1)	(250 m range)	
Model 1	‐‐‐‐	‐3.85*	13.86**	‐‐‐‐
Model 2	n.s.	‐3.93*	15.57*	‐‐‐‐
**SBV**				(infected)
Model 1	‐‐‐‐	‐‐‐‐	10.60** (250 m range)	8.70**
Model 2	‐‐‐‐	‐‐‐‐	8.73* (500 m range)	8.10**

*Note:* For each predictor included in a model we present the sign and magnitude of its scaled coefficient, and statistical significance at **p < 0.01; *p < 0.05; Statistically non‐significant predictors included in the models are indicated as n.s., and excluded predictors are indicated with dashes; We indicate in parentheses the best‐fitting range of the landscape predictor, and correspondence analysis (CA) axis, and the measure of honey bee density (total or infected only).

**FIGURE 2:**
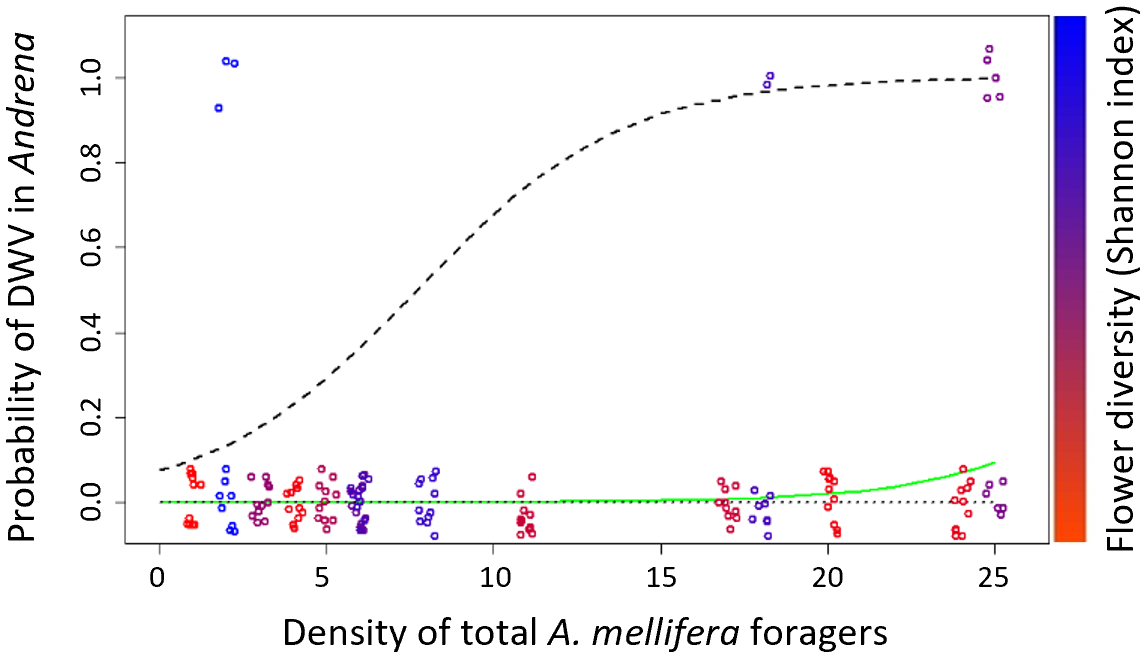
Selected model for the probability of deformed wing virus (DWV) presence in mining (*Andrena*) bees, as a function of total *A. mellifera* forager density and flower diversity (Shannon index) in the site. The lines represent model fits with the mean (green line) and maximum and minimum (dashed lines) flower diversity recorded in each site. Data points represent individual mining bees, with (1) or without (0) detected DWV, plotted according to the density of total *A. mellifera* foragers and colored according to the flower diversity at the sites. Points were slightly jittered (i.e., their positions were randomly modified to reduce overplotting).

A single model was selected for the probability of DWV presence in mining bees, describing a positive association with both floral diversity and total density of *A. mellifera* foragers (both DWV‐positive and negative; Table 1; Figure 2). Although the coefficient signs and significance of these predictors were stable in a perturbation analysis, their relative magnitudes were variable and negatively correlated due to mild collinearity. Thus, our data cannot disentangle the relative magnitudes of the association of floral diversity and honey bee density with DWV prevalence. Both landscape variables (FRA score and the proportion of non‐agricultural land) in the tested models did not have a significant association with the probability of DWV in mining bees (Table 1). Floral diversity was positively associated with total visitation density on yellow mustard plants (*p*‐value = 0.063).

Two similar models were selected for the probability of LSV‐2 presence in mining bees, including an association with the floral composition (1st CA axis) in the site and a positive association with FRA score within 250 m of the site (Table 1).

**FIGURE 3:**
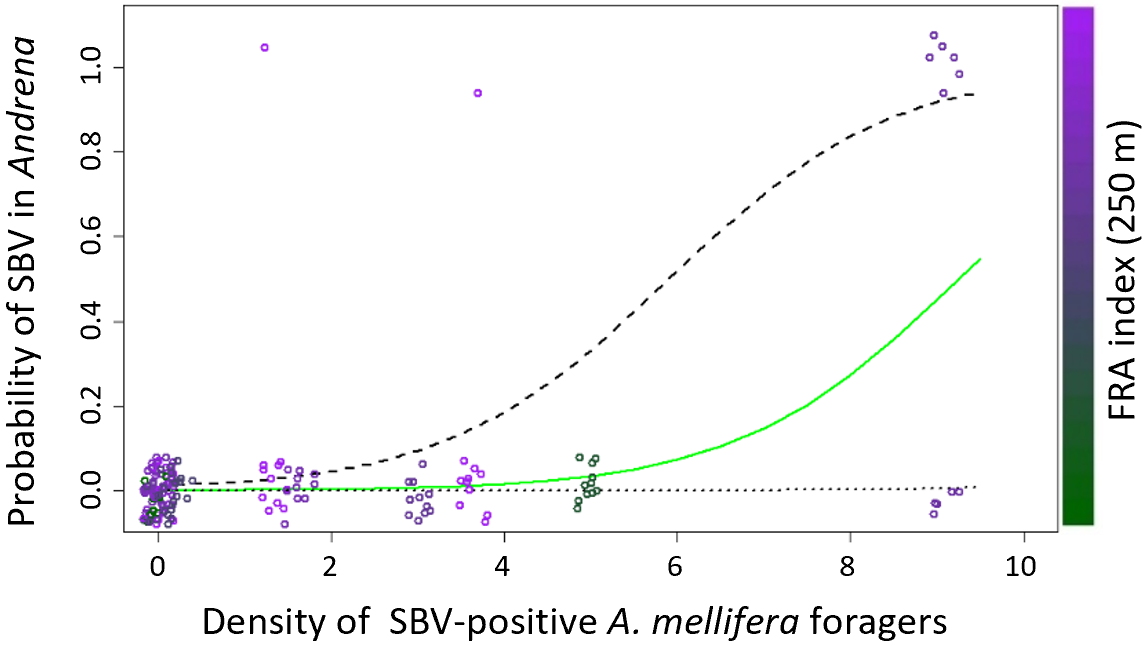
Selected model for the probability of sacbrood virus (SBV) presence in mining (*Andrena*) bees, as a function of SBV‐positive *A. mellifera* forager density in the site and floral resource availability (FRA) index within 250‐m range. The lines represent model fits, with the mean (green line) and maximum and minimum (dashed lines) FRA index of each site. Data points represent individual mining bees, with (1) or without (0) detected SBV, plotted according to the density of SBV‐positive *A. mellifera* foragers in the site and colored according to the level of FRA index at 250 m around the site. Points were slightly jittered (i.e., their positions were randomly modified to reduce overplotting).

Two similar models were selected for the probability of SBV presence in mining bees, with a positive association with the estimated density of SBV‐carrying *A. mellifera* foragers and with FRA scores within 250 or 500 m from the site (Table 1; Figure 3). Comparison of the SBV viral genome sequence obtained from the two bee taxa at the site with the highest prevalence of SBV‐positive *Andrena* (site name: R.B.Sh) revealed 98.3% nucleotide identity, indicating that SBV is transmitted between honey bees and mining bees in the surveyed site (GenBank accession numbers for either the *A. mellifera* and *Andrena* sequences are OQ994751 and OQ994752, respectively).

In order to explore the role of specific flower taxa on the associations found between flower composition and virus prevalence (BQCV and LSV‐2 in *Andrena* and BQCV in *A. mellifera*), we used the flower species CA scores. To distinguish specific flower taxa of potentially high effect on virus spread, we identified flower species that showed consistent association with the trends of both CA axes (Appendix S3). Among the flower taxa that comprised the CA axes and had substantial relative abundance across sites, *Echium judaeum* and *Notobasis syriaca* were positively correlated with CA1 and negatively correlated with CA2. Thus, these species were negatively associated with both LSV‐2 and BQCV prevalence in mining bees. Contrastingly, *Erucaria hispanica* was negatively correlated with CA1 and positively correlated with CA2. Accordingly, it was positively associated with both LSV‐2 and BQCV prevalence in mining bees.

## DISCUSSION

The prevalence of different viruses in mining bees is associated with all three groups of predictors that we explored, namely, (1) the local floral community, (2) FRA in the surrounding landscape (within up to 1000 m), and to a somewhat lesser degree (3) the local density of co‐foraging honey bees. Varying patterns and trends were found among the different viruses, possibly reflecting virus‐specific variation in the underlying transmission mechanisms. These mechanisms might include bee foraging ranges and behaviors, specific modes by which different viruses are deposited on or acquired from flowers by visiting bees, and how different viruses interact with their bee hosts. However, several broader patterns emerge from the results.

### The local floral community

We found that the local floral community, including species diversity and composition, was a broadly significant predictor for the presence of three out of the four viruses detected in mining bees (BQCV, DWV, and LSV‐2). The role of floral composition may be related to the potential of specific plant taxa to act as virus transmission hubs, depending on their floral traits (reviewed by Adler et al., 2020); for example, open and exposed flowers intercept higher solar radiation, which impairs pathogen viability and accelerates degradation, ultimately leading to shorter infection opportunities for subsequent visitors (Figueroa et al., 2019; Schmid‐Hempel, 1998). In addition, variation in floral composition and diversity alters the foraging behavior of pollinators (Buchmann, 1985; Russell et al., 2017) and thus may affect the spread of viruses in pollinator communities, as different foraging behaviors (e.g., nectaring, scrabbling) contribute differently to the deposition and acquisition of pathogens in flowers (Russell et al., 2019). Variation in flower composition may also alter the foraging preferences of pollinators; many bee species show context‐dependent foraging preferences, that vary considerably in space and time (e.g. CaraDonna et al., 2017; Cnaani et al., 2006; Goulson, 1999; Heinrich, 1976). This in turn may affect plant–pollinator networks and hence pathogen transmission through shared flowers (Tuerlings et al., 2022). In our study, variation in flower composition among the surveyed sites was significantly associated with the prevalence of BQCV and of LSV‐2 in mining bees. Specific flower species may alter the degree of overlap in floral resource use between pollinators and consequently opportunities for interspecific virus transmission. Since the focal *Andrena* species utilized the yellow mustard plants almost exclusively (~99% of observed visits; Appendix S3: Figure S1), variation in flower composition likely affected the foraging activity of sympatric polylectic bees on these yellow mustard plants. Specifically, sharing of yellow mustard flowers may have decreased in sites with greater relative abundance of other, more attractive, flower species. Additional study is required to further investigate and support the data presented herein regarding the role of specific flower taxa on virus prevalence in mining bees.

Floral diversity was positively related to DWV presence in mining bees; this is inconsistent with the general hypothesis that flower diversity dilutes pathogen spread due to increased modularity of plant–pollinator interactions and the subsequent reduction of interspecific resource sharing (Proesmans et al., 2021). However, higher flower diversity may sometimes lead to modifications in the pollination network (Morrison et al., 2020; Traveset et al., 2018; Vanbergen et al., 2014), creating new links between pollinator species whose diets are otherwise nonoverlapping (Cusser & Goodell, 2013). This may potentially introduce novel pathways of interspecific transmission (Figueroa et al., 2020) and increase the overall probability of virus transmission. Furthermore, an increase in flower diversity and pollinator richness can lead to asymmetric specialization (Vázquez & Aizen, 2004), contrasting the modularity effect of complex plant–pollinator networks. In our study system, besides the three dominant yellow mustard plants, *Erucaria hispanica* was the only flower species on which we observed visits by both the focal *Andrena* species and other sympatric bee species (mostly five species of *Eucera* and also honey bees) (see Appendix S3: Figure S1) and thus might create a new route for virus transmission in the sites at which it occurred. This is supported by the association of this flower species with higher prevalence of both BQCV and LSV‐2 in mining bees (see Appendix S3: Table S1). Variation in floral diversity, by altering flower–pollinator interactions, may also affect pollinator visitation density for each floral taxon. Specifically, we found that the visitation density of all bee species on the yellow mustard plants was positively associated with floral diversity, suggesting a higher probability of virus transmission due to flower sharing (reduced dilution by abundance) in sites with higher floral diversity.

### The surrounding landscape

The FRA score, reflecting FRA in the surrounding landscape, was also found to be a broadly significant predictor for the presence of most hymenopteran viruses detected in this study (BQCV, LSV‐2, and SBV in mining bees). Assessing landscape effects on pollinators is often done by categorical land‐use classification (Gallant et al., 2014). This approach was used by Figueroa et al. (2020), who detected a moderate effect of landscape simplification, quantified as the proportion of agricultural cover, on pathogen prevalence in bumble bees. Herein, the FRA score significantly outperformed the proportion of non‐agricultural land as a landscape‐level predictor (Appendix S3: Table S1). Thus, FRA may be a focal characteristic of the landscape that is of key importance for pollinator health, even for bees such as *Andrena* that have very limited foraging ranges (Gathmann & Tscharntke, 2002).

We found that FRA was positively related to the prevalence of three out of the four viruses detected in mining bees. A possible explanation might be that higher availability of floral resources is often associated with greater pollinator richness and abundance (Del Toro & Ribbons, 2020), which in turn may increase the richness and abundance of their viruses and other pathogens (Johnson et al., 2015). The introduction of these viruses from the surrounding landscape to the local population of mining bees, characterized by limited flight range, might occur via far‐dispersing individuals, or by far‐foraging honey bees (Beekman & Ratnieks, 2000), consistent with significant positive effects of SBV‐positive honey bee densities on SBV prevalence in mining bees. Taking into consideration these suggested mechanisms of virus spread, it is likely that varying life history and behavioral characteristics of different bee taxa will affect virus spread at different spatial scales.

Overall, the association of floral community characteristics with virus prevalence appears much stronger at the landscape scale than at the local scale, with up to one order of magnitude difference in the sizes of standardized coefficients (Table 1). This pattern implies that the ecological and epidemiological processes that impact virus spread among bee communities may be occurring at large spatial scales.

### The co‐foraging honey bees

In this survey, the densities of co‐foraging honey bees were positively related to the presence of two viruses, DWV and SBV, in mining bees. Previous phylogenetic studies have demonstrated sharing of SBV (Dolezal et al., 2016; Murray et al., 2019; Ravoet et al., 2014) as well as DWV (Murray et al., 2019; Singh et al., 2010) between sympatric honey bees and mining bees. Here, SBV prevalence was associated with the density of SBV‐positive honey bees, as expected if SBV is transmitted from honey bees to mining bees. In addition, the SBV genome sequence from mining bees and honey bees was 98.3% identical at the nucleotide level, indicating virus sharing between the two bee taxa. Prevalence of DWV was associated with the density of all honey bee foragers (both positive and negative for DWV). Honey bees might modulate the susceptibility of wild bees to viruses, by exerting competitive pressure (the facilitation hypothesis; Graystock et al., 2016). For example, honey bees could alter resource availability for wild bees, causing nutritional stress, or they could alter pollination networks, increasing resource sharing between DWV‐carrying and DWV‐free wild bee species (Magrach et al., 2017). However, we do not have any direct data to further explore this hypothesis. Due to mild multicollinearity, our data could not disentangle the relative magnitudes of honey bee density and floral species diversity in their association with DWV prevalence. Nevertheless, as DWV, BQCV, and LSV (LSV‐NE) sequence data of mining bees and honey bees from the same study sites show high nucleotide identity (virus‐augmented libraries shared 97%, 96%, and 97% nucleotide identity respectively; Daughenbaugh et al., 2021), it is likely that those viruses are also shared between honey bees and mining bees, but due to the dynamic/temporal nature of virus prevalence in bees (Graystock et al., 2020), these interspecific associations were not detected in our models. Taken together, direct support of virus transmission between honey bees and wild bees in this study is limited only to SBV. Taking into consideration the suggested mechanisms of virus transmission, it is likely that patterns of virus spread will exhibit similarity among pollinators sharing analogous life history and behavioral characteristics. Virus prevalence here was based on an epidemiological population of four species of *Andrena* whose foraging preferences and behavior, as well as life histories are very similar. Although species‐specific differences in immunity and physiological response to viral infection may have biological significance for viral replication rate and bee longevity, species‐specific differences in prevalence, which reflect the probability of bees acquiring viruses, were not detected in our sample of *Andrena*.

Although virus prevalence in *Andrena* bees was relatively low across all sites, this pattern is consistent with previous studies reporting similarly low rates in solitary bees (e.g., Dolezal et al., 2016; Murray et al., 2019; Radzevičiūtė et al., 2017). These low rates likely reflect inherent epidemiological and behavioral differences between these bee taxa—most notably the solitary lifestyle and short lifespan of *Andrena* bees, which limits direct contact between individuals and reduces opportunities for virus transmission. Still, even with infrequent detections, our use of presence–absence models—well suited to analyzing pathogen occurrence in low‐prevalence systems (Brotons et al., 2004; Zeimes et al., 2012)—allowed us to identify ecological associations with floral and landscape variables. These models draw information from both detections and non‐detections, and negative observations can meaningfully contribute to inference, especially when detection rates are low (Brauer et al., 2012).

Regarding interspecific transmission, the consistent detection of viruses in both honey bees and mining bees at the same sites, combined with high sequence similarity in the detected viruses, strongly supports the possibility of interspecific viral transmission (at least in the case of SBV). However, the lower prevalence in *Andrena* bees suggests that transmission from honey bees may occur only under specific ecological conditions or with low efficiency. Further research, including experimental or longitudinal studies, is needed to better quantify the frequency and mechanisms of interspecific virus transmission between co‐foraging bee species.

## CONCLUSIONS

Our results indicate that floral communities play a critical role in the spread of viruses in pollinator communities. Importantly, we found that these assumed floral‐mediated patterns of virus spread are present at both local and landscape scales and that the latter are better represented by the FRA index, incorporating spatiotemporal availability of resources, than by mere land‐use classification. The hypothesis that floral diversity mitigates virus spread within the pollinator community by a dilution effect is not supported for the viruses and bee taxa described herein, for which other mechanisms related to floral diversity and composition may have a more significant role. As flowers seem to be a key element in the process of interspecific virus spread in pollinator communities, research efforts should be directed at expanding our understanding of the role of flowers as virus transmission hubs at all levels, from individual flower traits to community‐level characteristics. In addition, temporal monitoring studies are needed to better assess the factors influencing virus transmission between co‐foraging bee species. Pollinator health programs should thus address flower communities, as both forage resources and possible hubs for virus spread, and apply a landscape perspective. Finally, pollinator conservation schemes should account for the possible spread of viruses between managed and native pollinator populations.

## AUTHOR CONTRIBUTIONS

Yael Mandelik, Asaf Sadeh, Idan Kahnonitch, Michelle L. Flenniken, and Nor Chejanovsky conceived the ideas and designed the methodology. Idan Kahnonitch and Na'ama Arkin conducted the field survey and collected the data. Achik Dorchin, together with Idan Kahnonitch and Naama Arkin, conducted the field‐ and lab‐taxonomic identification of the collected bees. Michelle L. Flenniken, Nor Chejanovsky, Katie F. Daughenbaugh, and Tal Erez conducted the high‐throughput sequencing and virus screening (qPCR) assays. Michelle L. Flenniken conducted the SBV comparative genomic analysis. Idan Kahnonitch analyzed the data. Idan Kahnonitch and Yael Mandelik led the writing of the manuscript. All authors contributed critically to the drafts and gave final approval for publication.

## CONFLICT OF INTEREST STATEMENT

The authors declare no conflicts of interest.

## Supporting information


Appendix S1.

Appendix S2.

Appendix S3.

Appendix S4.

Appendix S5.

## Data Availability

Data (Kahnonitch et al., 2025) is available in Dryad at https://doi.org/10.5061/dryad.crjdfn3df. SBV sequences are available in GenBank as follows: *A. mellifera*, accession no. OQ994751, https://www.ncbi.nlm.nih.gov/nuccore/OQ994751.1/; *Andrena*, accession no. OQ994752, https://www.ncbi.nlm.nih.gov/nuccore/OQ994752.

## References

[eap70133-bib-0001] Adler, L. S. , R. E. Irwin , S. H. McArt , and R. L. Vannette . 2020. “Floral Traits Affecting the Transmission of Beneficial and Pathogenic Pollinator‐Associated Microbes.” Current Opinion in Insect Science 44: 1–7. 32866657 10.1016/j.cois.2020.08.006 PMC7914268

[eap70133-bib-0002] Aizen, M. A. , and L. D. Harder . 2009. “The Global Stock of Domesticated Honey Bees Is Growing Slower than Agricultural Demand for Pollination.” Current Biology 19(11): 915–918. 19427214 10.1016/j.cub.2009.03.071

[eap70133-bib-0003] Akaike, H. 1987. “Factor Analysis and AIC.” In Selected Papers of Hirotugu Akaike 371–386. New York: Springer.

[eap70133-bib-0004] Alger, S. A. , P. A. Burnham , H. F. Boncristiani , and A. K. Brody . 2019. “RNA Virus Spillover from Managed Honeybees (*Apis mellifera*) to Wild Bumblebees (*Bombus* spp.).” PLoS One 14(6): e0217822. 31242222 10.1371/journal.pone.0217822 PMC6594593

[eap70133-bib-0005] Alger, S. A. , P. A. Burnham , and A. K. Brody . 2019. “Flowers as Viral Hot Spots: Honey Bees (*Apis mellifera*) Unevenly Deposit Viruses across Plant Species.” PLoS One 14(9): e0221800. 31532764 10.1371/journal.pone.0221800 PMC6750573

[eap70133-bib-0006] Babyak, M. A. 2009. “Rescaling continuous predictors in regression.” Statistical Tips from the Editors of Psychosomatic Medicine blog. Available at: http://stattips.blogspot.com/2009/08/rescaling-continuous-predictors-in.html .

[eap70133-bib-0007] Bartomeus, I. , J. S. Ascher , J. Gibbs , B. N. Danforth , D. L. Wagner , S. M. Hedtke , and R. Winfree . 2013. “Historical Changes in Northeastern US Bee Pollinators Related to Shared Ecological Traits.” Proceedings of the National Academy of Sciences 110(12): 4656–4660. 10.1073/pnas.1218503110 PMC3606985 23487768

[eap70133-bib-0008] Bates, D. , M. Maechler , S. Walker , R. Christensen , H. Singmann , B. Dai , and F. Scheipl . 2020. “Package ‘lme4’.” https://cran.r-project.org/web/packages/lme4/lme4.pdf.

[eap70133-bib-0009] Beekman, M. , and F. L. W. Ratnieks . 2000. “Long‐Range Foraging by the Honey‐Bee, *Apis mellifera* L.” Functional Ecology 14(4): 490–496.

[eap70133-bib-0010] Belsley, D. A. 1991. Conditioning Diagnostics: collinearity and Weak Data in Regression. New York: John Wiley and Sons.

[eap70133-bib-0011] Brauer, F. , C. Castillo‐Chavez , and C. Castillo‐Chavez . 2012. Mathematical Models in Population Biology and Epidemiology. New York: Springer.

[eap70133-bib-0012] Brooks, M. E. , K. Kristensen , K. J. Van Benthem , A. Magnusson , C. W. Berg , A. Nielsen , H. J. Skaug , M. Mächler , and B. M. Bolker . 2017. “glmmTMB Balances Speed and Flexibility among Packages for Zero‐Inflated Generalized Linear Mixed Modeling.” The R Journal 9(2): 378–400.

[eap70133-bib-0013] Brotons, L. , W. Thuiller , M. B. Araújo , and A. H. Hirzel . 2004. “Presence‐Absence Versus Presence‐Only Modelling Methods for Predicting Bird Habitat Suitability.” Ecography 27(4): 437–448.

[eap70133-bib-0014] Bruckner, S. , M. Wilson , D. Aurell , K. Rennich , D. Vanengelsdorp , N. Steinhauer , and G. R. Williams . 2023. “A National Survey of Managed Honey Bee Colony Losses in the USA: Results from the Bee Informed Partnership for 2017–18, 2018–19, and 2019–20.” Journal of Apicultural Research 62(3): 429–443.

[eap70133-bib-0015] Buchmann, S. L. 1985. “Bees Use Vibration to Aid Pollen Collection from Non‐Poricidal Flowers.” Journal of the Kansas Entomological Society 58(3): 517–525.

[eap70133-bib-0016] Burnham, P. A. , S. A. Alger , B. Case , H. Boncristiani , L. Hébert‐Dufresne , and A. K. Brody . 2021. “Flowers as Dirty Doorknobs: Deformed Wing Virus Transmitted between *Apis mellifera* and *Bombus impatiens* through Shared Flowers.” Journal of Applied Ecology 58(10): 2065–2074.

[eap70133-bib-0017] Cane, J. H. , and S. Sipes . 2006. “Characterizing Floral Specialization by Bees: analytical Methods and a Revised Lexicon for Oligolecty.” Plant‐Pollinator Interactions: From Specialization to Generalization 99: 122.

[eap70133-bib-0018] CaraDonna, P. J. , W. K. Petry , R. M. Brennan , J. L. Cunningham , J. L. Bronstein , N. M. Waser , and N. J. Sanders . 2017. “Interaction Rewiring and the Rapid Turnover of Plant–Pollinator Networks.” Ecology Letters 20(3): 385–394. 28156041 10.1111/ele.12740

[eap70133-bib-0019] Carvalheiro, L. G. , W. E. Kunin , P. Keil , J. Aguirre‐Gutiérrez , W. N. Ellis , R. Fox , Q. Groom , S. Hennekens , W. van Landuyt , and D. Maes . 2013. “Species Richness Declines and Biotic Homogenisation Have Slowed Down for NW‐European Pollinators and Plants.” Ecology Letters 16(7): 870–878. 23692632 10.1111/ele.12121 PMC3738924

[eap70133-bib-0020] Chen, Y. , J. Evans , and M. Feldlaufer . 2006. “Horizontal and Vertical Transmission of Viruses in the Honey Bee, *Apis mellifera* .” Journal of Invertebrate Pathology 92(3): 152–159. 16793058 10.1016/j.jip.2006.03.010

[eap70133-bib-0021] Cnaani, J. , J. D. Thomson , and D. R. Papaj . 2006. “Flower Choice and Learning in Foraging Bumblebees: Effects of Variation in Nectar Volume and Concentration.” Ethology 112(3): 278–285.

[eap70133-bib-0022] Cusser, S. , and K. Goodell . 2013. “Diversity and Distribution of Floral Resources Influence the Restoration of Plant–Pollinator Networks on a Reclaimed Strip Mine.” Restoration Ecology 21(6): 713–721.

[eap70133-bib-0023] Daughenbaugh, K. F. , I. Kahnonitch , C. C. Carey , A. J. McMenamin , T. Wiegand , T. Erez , N. Arkin , et al. 2021. “Metatranscriptome Analysis of Sympatric Bee Species Identifies Bee Virus Variants and a New Virus, Andrena‐Associated Bee Virus‐1.” Viruses 13(2): 291. 33673324 10.3390/v13020291 PMC7917660

[eap70133-bib-0024] Daughenbaugh, K. F. , M. Martin , L. M. Brutscher , I. Cavigli , E. Garcia , M. Lavin , and M. L. Flenniken . 2015. “Honey Bee Infecting Lake Sinai Viruses.” Viruses 7(6): 3285–3309. 26110586 10.3390/v7062772 PMC4488739

[eap70133-bib-0025] De Miranda, J. R. , L. Bailey , B. V. Ball , P. Blanchard , G. E. Budge , N. Chejanovsky , Y. Chen , et al. 2013. “Standard Methods for Virus Research in *Apis mellifera* .” Journal of Apicultural Research 52(4): 1–56.

[eap70133-bib-0026] Del Toro, I. , and R. R. Ribbons . 2020. “No Mow May Lawns Have Higher Pollinator Richness and Abundances: An Engaged Community Provides Floral Resources for Pollinators.” PeerJ 8: e10021. 33024642 10.7717/peerj.10021 PMC7518183

[eap70133-bib-0027] Dobelmann, J. , A. Felden , and P. J. Lester . 2020. “Genetic Strain Diversity of Multi‐Host RNA Viruses that Infect a Wide Range of Pollinators and Associates Is Shaped by Geographic Origins.” Viruses 12(3): 358. 32213950 10.3390/v12030358 PMC7150836

[eap70133-bib-0028] Dolezal, A. G. , S. D. Hendrix , N. A. Scavo , J. Carrillo‐Tripp , M. A. Harris , M. J. Wheelock , M. E. O'Neal , and A. L. Toth . 2016. “Honey Bee Viruses in Wild Bees: viral Prevalence, Loads, and Experimental Inoculation.” PLoS One 11(11): e0166190. 27832169 10.1371/journal.pone.0166190 PMC5104440

[eap70133-bib-0029] Figueroa, L. L. , M. Blinder , C. Grincavitch , A. Jelinek , E. K. Mann , L. A. Merva , L. E. Metz , et al. 2019. “Bee Pathogen Transmission Dynamics: Deposition, Persistence and Acquisition on Flowers.” Proceedings of the Royal Society B 286(1903): 20190603. 31138075 10.1098/rspb.2019.0603 PMC6545085

[eap70133-bib-0030] Figueroa, L. L. , H. Grab , W. H. Ng , C. R. Myers , P. Graystock , Q. S. McFrederick , and S. H. McArt . 2020. “Landscape Simplification Shapes Pathogen Prevalence in Plant‐Pollinator Networks.” Ecology Letters 23(8): 1212–1222. 32347001 10.1111/ele.13521 PMC7340580

[eap70133-bib-0031] Fox, J. , S. Weisberg , D. Adler , D. Bates , G. Baud‐Bovy , S. Ellison , and D. Firth . 2012. “Package ‘Car’.” R Package Version 2.1–6. https://cran.r-project.org/web/packages/car/

[eap70133-bib-0032] Fürst, M. A. , D. P. McMahon , J. L. Osborne , R. J. Paxton , and M. J. F. Brown . 2014. “Disease Associations between Honeybees and Bumblebees as a Threat to Wild Pollinators.” Nature 506(7488): 364–366. 24553241 10.1038/nature12977 PMC3985068

[eap70133-bib-0033] Gallant, A. L. , N. H. Euliss, Jr. , and Z. Browning . 2014. “Mapping Large‐Area Landscape Suitability for Honey Bees to Assess the Influence of Land‐Use Change on Sustainability of National Pollination Services.” PLoS One 9(6): e99268. 24919181 10.1371/journal.pone.0099268 PMC4053381

[eap70133-bib-0034] Garibaldi, L. A. , D. S. Gomez Carella , D. N. Nabaes Jodar , M. R. Smith , T. P. Timberlake , and S. S. Myers . 2022. “Exploring Connections between Pollinator Health and Human Health.” Philosophical Transactions of the Royal Society B 377(1853): 20210158. 10.1098/rstb.2021.0158 PMC9058530 35491592

[eap70133-bib-0035] Gathmann, A. , and T. Tscharntke . 2002. “Foraging Ranges of Solitary Bees.” Journal of Animal Ecology 71(5): 757–764.

[eap70133-bib-0036] Genersch, E. 2010. “Honey Bee Pathology: Current Threats to Honey Bees and Beekeeping.” Applied Microbiology and Biotechnology 87(1): 87–97. 20401479 10.1007/s00253-010-2573-8

[eap70133-bib-0037] Goulson, D. 1999. “Foraging Strategies of Insects for Gathering Nectar and Pollen, and Implications for Plant Ecology and Evolution.” Perspectives in Plant Ecology, Evolution and Systematics 2(2): 185–209.

[eap70133-bib-0038] Goulson, D. , and W. O. Hughes . 2015. “Mitigating the Anthropogenic Spread of Bee Parasites to Protect Wild Pollinators.” Biological Conservation 191: 10–19.

[eap70133-bib-0039] Graystock, P. , E. J. Blane , Q. S. McFrederick , D. Goulson , and W. O. Hughes . 2016. “Do Managed Bees Drive Parasite Spread and Emergence in Wild Bees?” International Journal for Parasitology: Parasites and Wildlife 5(1): 64–75. 28560161 10.1016/j.ijppaw.2015.10.001 PMC5439461

[eap70133-bib-0103] Graystock, P. , W. H. Ng , K. Parks , A. D. Tripodi , P. A. Muñiz , A. A. Fersch , C. R. Myers , Q. S. McFrederick , and S. H. McArt . 2020. “Dominant Bee Species and Floral Abundance Drive Parasite Temporal Dynamics in Plant‐Pollinator Communities.” Nature Ecology & Evolution 4(10): 1358–1367. 32690902 10.1038/s41559-020-1247-x PMC7529964

[eap70133-bib-0040] Grozinger, C. M. , and M. L. Flenniken . 2019. “Bee Viruses: Ecology, Pathogenicity, and Impacts.” Annual Review of Entomology 64: 205–226. 10.1146/annurev-ento-011118-111942 30629896

[eap70133-bib-0041] Hartig, F. , and L. Lohse . 2022. “DHARMa: Residual Diagnostics for Hierarchical (Multi‐Level/Mixed) Regression Models.” Version 0.4.6. https://cran.r-project.org/package=DHARMa

[eap70133-bib-0042] Heinrich, B. 1976. “The Foraging Specializations of Individual Bumblebees.” Ecological Monographs 46(2): 105–128.

[eap70133-bib-0043] Johnson, P. T. , R. S. Ostfeld , and F. Keesing . 2015. “Frontiers in Research on Biodiversity and Disease.” Ecology Letters 18(10): 1119–1133. 26261049 10.1111/ele.12479 PMC4860816

[eap70133-bib-0044] Kahnonitch, I. , Y. Mandelik , K. F. Daughenbaugh , N. Arkin , T. Erez , A. Dorchin , M. L. Flenniken , N. Chejanovsky , and A. Sadeh . 2025. “Primers, Site Data, and Virus Abundance in Honey Bees and *Andrena* spp.” Dryad. [Dataset] 10.5061/dryad.crjdfn3df

[eap70133-bib-0045] Klein, A. M. , B. E. Vaissière , J. H. Cane , I. Steffan‐Dewenter , S. A. Cunningham , C. Kremen , and T. Tscharntke . 2007. “Importance of Pollinators in Changing Landscapes for World Crops.” Proceedings of the Royal Society B: Biological Sciences 274(1608): 303–313. 10.1098/rspb.2006.3721 PMC1702377 17164193

[eap70133-bib-0046] Kline, O. , N. T. Phan , M. F. Porras , J. Chavana , C. Z. Little , L. Stemet , R. S. Acharya , D. J. Biddinger , G. V. P. Reddy , and N. K. Joshi . 2023. “Biology, Genetic Diversity, and Conservation of Wild Bees in Tree Fruit Orchards.” Biology 12: 31. 10.3390/biology12010031 PMC9854918 36671724

[eap70133-bib-0047] Kremen, C. , N. M. Williams , M. A. Aizen , B. Gemmill‐Herren , G. LeBuhn , R. Minckley , L. Packer , et al. 2007. “Pollination and Other Ecosystem Services Produced by Mobile Organisms: A Conceptual Framework for the Effects of Land‐Use Change.” Ecology Letters 10(4): 299–314. 17355569 10.1111/j.1461-0248.2007.01018.x

[eap70133-bib-0048] Lauring, A. S. , and R. Andino . 2010. “Quasispecies Theory and the Behavior of RNA Viruses.” PLoS Pathogens 6(7): e1001005. 20661479 10.1371/journal.ppat.1001005 PMC2908548

[eap70133-bib-0049] Levenson, H. K. , and D. R. Tarpy . 2022. “Effects of Planted Pollinator Habitat on Pathogen Prevalence and Interspecific Detection between Bee Species.” Scientific Reports 12(1): 1–11. 35551218 10.1038/s41598-022-11734-3 PMC9098541

[eap70133-bib-0050] Lonsdorf, E. , C. Kremen , T. Ricketts , R. Winfree , N. Williams , and S. Greenleaf . 2009. “Modelling Pollination Services Across Agricultural Landscapes.” Annals of Botany 103(9): 1589–1600. 19324897 10.1093/aob/mcp069 PMC2701767

[eap70133-bib-0051] Lotan, A. , R. Kost , Y. Mandelik , Y. Peled , D. Chakuki , S. Z. Shamir , and Y. Ram . 2018. “National Scale Mapping of Ecosystem Services in Israel–Genetic Resources, Pollination and Cultural Services.” One Ecosystem 3: e25494.

[eap70133-bib-0052] Magrach, A. , J. P. González‐Varo , M. Boiffier , M. Vilà , and I. Bartomeus . 2017. “Honeybee Spillover Reshuffles Pollinator Diets and Affects Plant Reproductive Success.” Nature Ecology and Evolution 1(9): 1299–1307. 29046536 10.1038/s41559-017-0249-9

[eap70133-bib-0053] Manley, R. , M. Boots , and L. Wilfert . 2015. “Emerging Viral Disease Risk to Pollinating Insects: ecological, Evolutionary and Anthropogenic Factors.” Journal of Applied Ecology 52(2): 331–340. 25954053 10.1111/1365-2664.12385 PMC4415536

[eap70133-bib-0054] Mao, W. , M. A. Schuler , and M. R. Berenbaum . 2013. “Honey Constituents Up‐Regulate Detoxification and Immunity Genes in the Western Honey Bee *Apis mellifera* .” Proceedings of the National Academy of Sciences 110(22): 8842–8846. 10.1073/pnas.1303884110 PMC3670375 23630255

[eap70133-bib-0055] McArt, S. H. , H. Koch , R. E. Irwin , and L. S. Adler . 2014. “Arranging the Bouquet of Disease: Floral Traits and the Transmission of Plant and Animal Pathogens.” Ecology Letters 17(5): 624–636. 24528408 10.1111/ele.12257

[eap70133-bib-0056] McMahon, D. P. , M. A. Fürst , J. Caspar , P. Theodorou , M. J. Brown , and R. J. Paxton . 2015. “A Sting in the Spit: Widespread Cross‐Infection of Multiple RNA Viruses across Wild and Managed Bees.” Journal of Animal Ecology 84(3): 615–624. 25646973 10.1111/1365-2656.12345 PMC4832299

[eap70133-bib-0057] McNeil, D. J. , E. McCormick , A. C. Heimann , M. Kammerer , M. R. Douglas , S. C. Goslee , C. M. Grozinger , and H. M. Hines . 2020. “Bumble Bees in Landscapes with Abundant Floral Resources Have Lower Pathogen Loads.” Scientific Reports 10(1): 1–12. 33339846 10.1038/s41598-020-78119-2 PMC7749142

[eap70133-bib-0059] Michener, C. D. 1974. The Social Behavior of the Bees: a Comparative Study. Cambridge Massachusetts: Belknap Press of Harvard University Press.

[eap70133-bib-0060] Morrison, B. M. , B. J. Brosi , and R. Dirzo . 2020. “Agricultural Intensification Drives Changes in Hybrid Network Robustness by Modifying Network Structure.” Ecology Letters 23(2): 359–369. 31814265 10.1111/ele.13440

[eap70133-bib-0061] Murray, E. A. , J. Burand , N. Trikoz , J. Schnabel , H. Grab , and B. N. Danforth . 2019. “Viral Transmission in Honey Bees and Native Bees, Supported by a Global Black Queen Cell Virus Phylogeny.” Environmental Microbiology 21(3): 972–983. 30537211 10.1111/1462-2920.14501

[eap70133-bib-0062] Nanetti, A. , L. Bortolotti , and G. Cilia . 2021. “Pathogens Spillover from Honey Bees to Other Arthropods.” Pathogens 10(8): 1044. 34451508 10.3390/pathogens10081044 PMC8400633

[eap70133-bib-0063] Nazzi, F. , S. P. Brown , D. Annoscia , F. Del Piccolo , G. Di Prisco , P. Varricchio , G. D. Vedova , F. Cattonaro , E. Caprio , and F. Pennacchio . 2012. “Synergistic Parasite‐Pathogen Interactions Mediated by Host Immunity Can Drive the Collapse of Honeybee Colonies.” PLoS Pathogens 8(6): e1002735. 22719246 10.1371/journal.ppat.1002735 PMC3375299

[eap70133-bib-0064] Oksanen, J. , F. G. Blanchet , R. Kindt , P. Legendre , P. R. Minchin , R. B. O'Hara , G. L. Simpson , P. Solymos , M. H. H. Stevens , and H. Wagner . 2020. “vegan: Community Ecology Package.” R Package Version 2.5–6. https://CRAN.R-project.org/package=vegan

[eap70133-bib-0065] Ollerton, J. , R. Winfree , and S. Tarrant . 2011. “How Many Flowering Plants Are Pollinated by Animals?” Oikos 120(3): 321–326.

[eap70133-bib-0066] Pfeiffer, V. W. , and D. W. Crowder . 2022. “Factors Affecting Virus Prevalence in Honey Bees in the Pacific Northwest, USA.” Journal of Invertebrate Pathology 187: 107703. 34902395 10.1016/j.jip.2021.107703

[eap70133-bib-0067] Piot, N. , G. Smagghe , and I. Meeus . 2020. “Network Centrality as an Indicator for Pollinator Parasite Transmission via Flowers.” Insects 11(12): 872. 33302397 10.3390/insects11120872 PMC7762566

[eap70133-bib-0068] Pisanty, G. , A. M. Klein , and Y. Mandelik . 2014. “Do Wild Bees Complement Honeybee Pollination of Confection Sunflowers in Israel?” Apidologie 45(2): 235–247.

[eap70133-bib-0069] Pisanty, G. , and Y. Mandelik . 2015. “Profiling Crop Pollinators: life History Traits Predict Habitat Use and Crop Visitation by Mediterranean Wild Bees.” Ecological Applications 25(3): 742–752. 26214919 10.1890/14-0910.1

[eap70133-bib-0070] Pisanty, G. , E. Scheuchl , and N. Dorchin . 2016. “Eight New Species of *Andrena* Fabricius (Hymenoptera: Apoidea: Andrenidae) from Israel‐a Mediterranean Hotspot for Wild Bees.” Zootaxa 4189(3): zootaxa‐4189. 10.11646/zootaxa.4189.3.3 27988745

[eap70133-bib-0071] Pisanty, G. , E. Scheuchl , T. Martin , S. Cardinal , and T. J. Wood . 2022. “Twenty‐Five New Species of Mining Bees (Hymenoptera: Andrenidae: *Andrena*) from Israel and the Levant.” Zootaxa 5185(1): 1–109. 37044814 10.11646/zootaxa.5185.1.1

[eap70133-bib-0072] Popovska Stojanov, D. , L. Dimitrov , J. Danihlík , A. Uzunov , M. Golubovski , S. Andonov , and R. Brodschneider . 2021. “Direct Economic Impact Assessment of Winter Honeybee Colony Losses in Three European Countries.” Agriculture 11(5): 398.

[eap70133-bib-0073] Posada‐Florez, F. , Z. S. Lamas , D. J. Hawthorne , Y. Chen , J. D. Evans , and E. V. Ryabov . 2021. “Pupal Cannibalism by Worker Honey Bees Contributes to the Spread of Deformed Wing Virus.” Scientific Reports 11(1): 1–12. 33903723 10.1038/s41598-021-88649-y PMC8076318

[eap70133-bib-0074] Potts, S. G. , J. C. Biesmeijer , C. Kremen , P. Neumann , O. Schweiger , and W. E. Kunin . 2010. “Global Pollinator Declines: Trends, Impacts and Drivers.” Trends in Ecology and Evolution 25(6): 345–353. 20188434 10.1016/j.tree.2010.01.007

[eap70133-bib-0075] Proesmans, W. , M. Albrecht , A. Gajda , P. Neumann , R. J. Paxton , M. Pioz , C. Polzin , et al. 2021. “Pathways for Novel Epidemiology: Plant–Pollinator–Pathogen Networks and Global Change.” Trends in Ecology and Evolution 36(7): 623–636. 33865639 10.1016/j.tree.2021.03.006

[eap70133-bib-0076] R Core Team . 2022. R: A Language and Environment for Statistical Computing. Vienna: R Foundation for Statistical Computing. https://www.R-project.org/.

[eap70133-bib-0077] Radzevičiūtė, R. , P. Theodorou , M. Husemann , G. Japoshvili , G. Kirkitadze , A. Zhusupbaeva , and R. J. Paxton . 2017. “Replication of Honey Bee‐Associated RNA Viruses across Multiple Bee Species in Apple Orchards of Georgia, Germany and Kyrgyzstan.” Journal of Invertebrate Pathology 146: 14–23. 28392285 10.1016/j.jip.2017.04.002

[eap70133-bib-0078] Ravoet, J. , L. De Smet , I. Meeus , G. Smagghe , T. Wenseleers , and D. C. de Graaf . 2014. “Widespread Occurrence of Honey Bee Pathogens in Solitary Bees.” Journal of Invertebrate Pathology 122: 55–58. 25196470 10.1016/j.jip.2014.08.007

[eap70133-bib-0079] Ripley, B. , B. Venables , D. M. Bates , K. Hornik , A. Gebhardt , D. Firth , and M. B. Ripley . 2013. “Package ‘Mass’.” CRAN R 538: 113–120.

[eap70133-bib-0080] Roth, T. , M. Coll , and Y. Mandelik . 2023. “The Role of Uncultivated Habitats in Supporting Wild Bee Communities in Mediterranean Agricultural Landscapes.” Diversity 15(2): 294.

[eap70133-bib-0081] Ruiz‐González, M. X. , J. Bryden , Y. Moret , C. Reber‐Funk , P. Schmid‐Hempel , and M. J. Brown . 2012. “Dynamic Transmission, Host Quality, and Population Structure in a Multihost Parasite of Bumblebees.” Evolution 66(10): 3053–3066. 23025597 10.1111/j.1558-5646.2012.01655.x

[eap70133-bib-0082] Russell, A. L. , S. L. Buchmann , and D. R. Papaj . 2017. “How a Generalist Bee Achieves High Efficiency of Pollen Collection on Diverse Floral Resources.” Behavioral Ecology 28(4): 991–1003.

[eap70133-bib-0083] Russell, A. L. , M. Rebolleda‐Gómez , T. M. Shaible , and T. L. Ashman . 2019. “Movers and Shakers: Bumble Bee Foraging Behavior Shapes the Dispersal of Microbes among and Within Flowers.” Ecosphere 10(5): e02714.

[eap70133-bib-0084] Schmid‐Hempel, P. 1998. Parasites in Social Insects. Princeton: Princeton University Press.

[eap70133-bib-0085] Schurr, L. , L. Affre , F. Flacher , T. Tatoni , L. Le Mire Pecheux , and B. Geslin . 2019. “Pollination Insights for the Conservation of a Rare Threatened Plant Species, *Astragalus tragacantha* (Fabaceae).” Biodiversity and Conservation 28: 1389–1409.

[eap70133-bib-0086] Singh, R. , A. L. Levitt , E. G. Rajotte , E. C. Holmes , N. Ostiguy , D. Vanengelsdorp , W. I. Lipkin , C. W. Depamphilis , A. L. Toth , and D. L. Cox‐Foster . 2010. “RNA Viruses in Hymenopteran Pollinators: evidence of Inter‐Taxa Virus Transmission Via Pollen and Potential Impact on Non‐*Apis* Hymenopteran Species.” PLoS One 5(12): e14357. 21203504 10.1371/journal.pone.0014357 PMC3008715

[eap70133-bib-0104] Sorek, M. , and I. Shapira , eds. 2018. Israel State of Nature report 2018. Tel Aviv University, Hamaarag, Steinhardt Museum of Natural History, Tel Aviv, Israel (In Hebrew).

[eap70133-bib-0087] Soroker, V. , Y. Slabezki , and N. Chejanovsky . 2018. “Apiculture in Israel.” In Asian beekeeping in the 21st century, edited by P. Chantawannakul , G. Williams , and P. Neumann , 95–109. Singapore: Springer.

[eap70133-bib-0088] Spivak, M. , and D. P. Cariveau . 2020. “Flowers as Parasite Transmission Hubs.” Nature Ecology and Evolution 4(10): 1298–1299. 32690903 10.1038/s41559-020-1200-z

[eap70133-bib-0089] Tang, J. , Q. M. Quan , J. Z. Chen , T. Wu , and S. Q. Huang . 2019. “Pollinator Effectiveness and Importance Between Female and Male Mining Bee (*Andrena*).” Biology Letters 15(10): 20190479. 31662065 10.1098/rsbl.2019.0479 PMC6832193

[eap70133-bib-0090] Tehel, A. , M. J. Brown , and R. J. Paxton . 2016. “Impact of Managed Honey Bee Viruses on Wild Bees.” Current Opinion in Virology 19: 16–22. 27351468 10.1016/j.coviro.2016.06.006

[eap70133-bib-0091] Traveset, A. , R. Castro‐Urgal , X. Rotllàn‐Puig , and A. Lázaro . 2018. “Effects of Habitat Loss on the Plant–Flower Visitor Network Structure of a Dune Community.” Oikos 127(1): 45–55.

[eap70133-bib-0092] Traynor, K. S. , K. Rennich , E. Forsgren , R. Rose , J. Pettis , G. Kunkel , and D. Vanengelsdorp . 2016. “Multiyear Survey Targeting Disease Incidence in US Honey Bees.” Apidologie 47: 325–347.

[eap70133-bib-0093] Tuerlings, T. , L. Buydens , G. Smagghe , and N. Piot . 2022. “The Impact of Mass‐Flowering Crops on Bee Pathogen Dynamics.” International Journal for Parasitology: Parasites and Wildlife 18: 135–147. 35586790 10.1016/j.ijppaw.2022.05.001 PMC9108762

[eap70133-bib-0094] Vanbergen, A. J. , B. A. Woodcock , A. Gray , F. Grant , A. Telford , P. Lambdon , D. S. Chapman , R. F. Pywell , M. S. Heard , and S. Cavers . 2014. “Grazing Alters Insect Visitation Networks and Plant Mating Systems.” Functional Ecology 28(1): 178–189.

[eap70133-bib-0095] vanEngelsdorp, D. , and M. D. Meixner . 2010. “A Historical Review of Managed Honey Bee Populations in Europe and the United States and the Factors That May Affect Them.” Journal of Invertebrate Pathology 103: S80–S95. 19909973 10.1016/j.jip.2009.06.011

[eap70133-bib-0096] Vázquez, D. P. , and M. A. Aizen . 2004. “Asymmetric Specialization: a Pervasive Feature of Plant–Pollinator Interactions.” Ecology 85(5): 1251–1257.

[eap70133-bib-0097] Wcislo, W. T. , and J. H. Cane . 1996. “Floral Resource Utilization by Solitary Bees (Hymenoptera: Apoidea) and Exploitation of their Stored Foods by Natural Enemies.” Annual Review of Entomology 41(1): 257–286. 10.1146/annurev.en.41.010196.001353 15012330

[eap70133-bib-0098] Wilfert, L. , G. Long , H. C. Leggett , P. Schmid‐Hempel , R. Butlin , S. J. M. Martin , and M. Boots . 2016. “Deformed Wing Virus Is a Recent Global Epidemic in Honeybees Driven by *Varroa* Mites.” Science 351(6273): 594–597. 26912700 10.1126/science.aac9976

[eap70133-bib-0099] Yañez, O. , N. Piot , A. Dalmon , J. R. de Miranda , P. Chantawannakul , D. Panziera , E. Amiri , G. Smagghe , D. Schroeder , and N. Chejanovsky . 2020. “Bee Viruses: Routes of Infection in Hymenoptera.” Frontiers in Microbiology 11: 943. 32547504 10.3389/fmicb.2020.00943 PMC7270585

[eap70133-bib-0100] Yañez, O. , H. Q. Zheng , X. L. Su , F. L. Hu , P. Neumann , and V. Dietemann . 2015. “Potential for Virus Transfer between the Honey Bees *Apis mellifera* and *A. cerana* .” Journal of Apicultural Research 54(3): 179–191.

[eap70133-bib-0101] Zeimes, C. B. , G. E. Olsson , C. Ahlm , and S. O. Vanwambeke . 2012. “Modelling Zoonotic Diseases in Humans: Comparison of Methods for Hantavirus in Sweden.” International Journal of Health Geographics 11: 1–12. 22984887 10.1186/1476-072X-11-39 PMC3517350

[eap70133-bib-0102] Zou, Y. , F. J. Bianchi , F. Jauker , H. Xiao , J. Chen , J. Cresswell , S. Luo , et al. 2017. “Landscape Effects on Pollinator Communities and Pollination Services in Small‐Holder Agroecosystems.” Agriculture, Ecosystems & Environment 246: 109–116.

