# Supplementary material for: Virus distributions in wild bees are associated with floral communities at local to landscape scales
Source: Ecol Appl. 2025 Nov 11;35(7):e70133. doi: 10.1002/eap.70133 (PMC12604080; doi:10.1002/eap.70133)
Supplement: Supplementary file 1 — Appendix S1. [file EAP-35-e70133-s005.pdf]

Virus distributions in wild bees are associated with floral communities at local to landscape scales

Idan Kahnonitch, Katie F. Daughenbaugh, Na'ama Arkin, Tal Erez, Achik Dorchin, Michelle L. Flenniken, Nor Chejanovsky, Asaf Sadeh, Yael Mandelik

Ecological Applications

Appendix S1

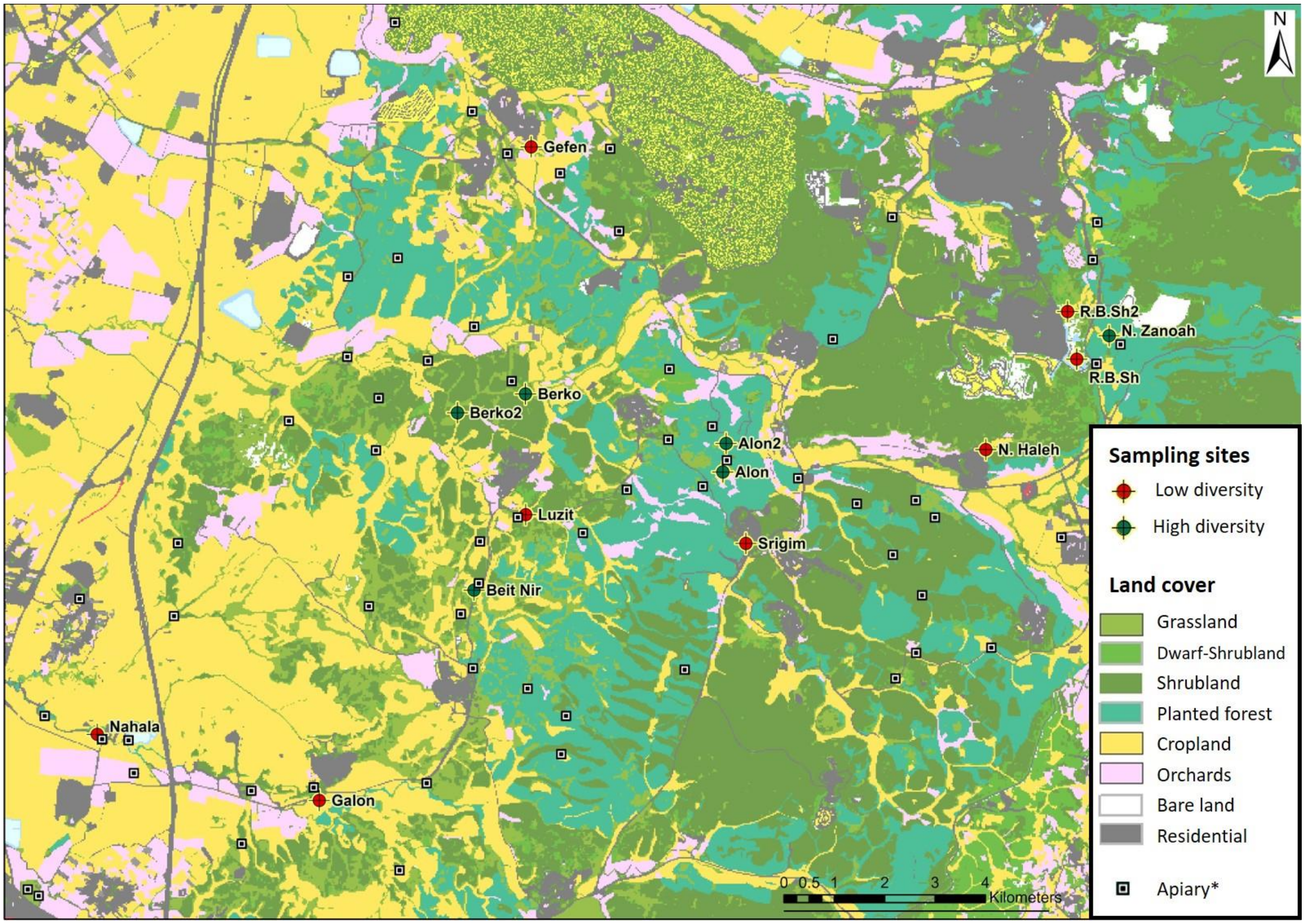

Figure S1. Map of the study area (Judea foothills, central Israel) with sampling sites (N=14) labeled and marked with green and red dots indicating high and low floral diversity, respectively, and surrounding land-cover. *White dotted squares indicate the location of apiaries within a radius of four kilometers around each sampling site.
