# Supplementary material for: Virus distributions in wild bees are associated with floral communities at local to landscape scales
Source: Ecol Appl. 2025 Nov 11;35(7):e70133. doi: 10.1002/eap.70133 (PMC12604080; doi:10.1002/eap.70133)
Supplement: Supplementary file 2 — Appendix S2. [file EAP-35-e70133-s002.pdf]

Virus distributions in wild bees are associated with floral communities at local to landscape scales

Idan Kahnonitch, Katie F. Daughenbaugh, Na'ama Arkin, Tal Erez, Achik Dorchin, Michelle L. Flenniken, Nor Chejanovsky, Asaf Sadeh, Yael Mandelik

Ecological Applications

Appendix S2

To evaluate whether zero inflation was a concern in our virus prevalence models, we conducted two diagnostic procedures:

1. Zero Inflation Test

We used the testZeroInflation function from the DHARMa package (Hartig & Lohse, 2022) to assess whether the observed number of zero responses significantly deviated from those expected under the fitted models. These tests were conducted separately for each virus model (SBV, LSV, BQCV, and DWV).

2. AICc Comparison (GLMM vs. Zero-Inflated Models)

To determine whether zero-inflated models provided a better fit to the data, we compared the AICc scores between standard GLMMs and their corresponding zero-inflated versions (ZIGLMMs), using the glmmTMB package (Brooks et al., 2017).

Results:

SBV:

- Zero Inflation Test: No indication of excess zeros (Figure S1A).
- Model comparison: GLMM AICc = 39.00; Zero-inflated model AICc = 41.14. The GLMM performed better.

BQCV:

- Zero Inflation Test: No indication of excess zeros (Figure S1B).
- Model comparison: GLMM AICc = 59.66; Zero-inflated model AICc = 60.46. The GLMM performed marginally better.

LSV:

- Zero Inflation Test: No indication of excess zeros (Figure S1C).
- Model comparison: GLMM AICc = 98.77; Zero-inflated model AICc = 100.17. The GLMM performed better.

DWV:

- Zero Inflation Test: No indication of excess zeros (Figure S1D).
- Model comparison: Fitting a zero-inflated model resulted in convergence issues and high multicollinearity (VIF = 12.14), making the model unreliable.

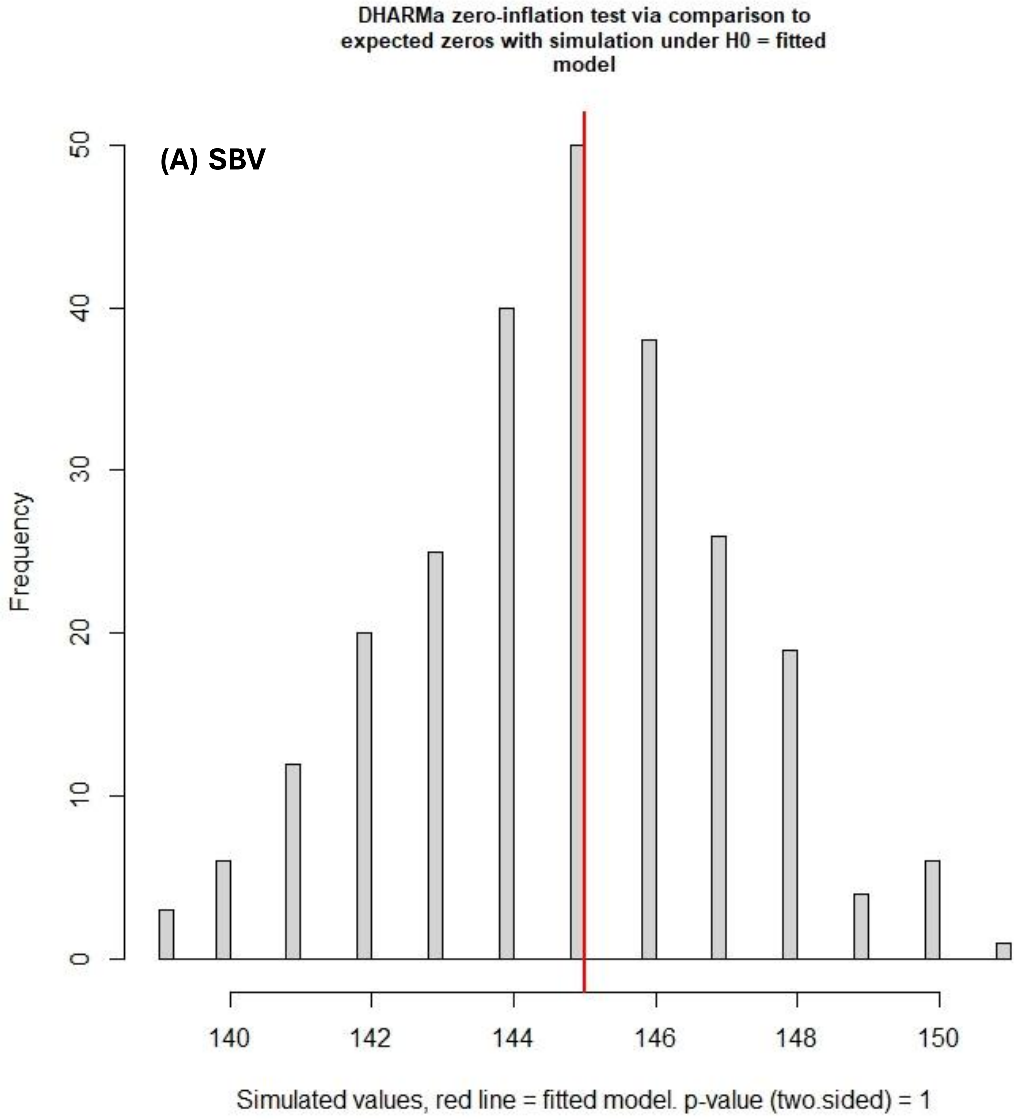

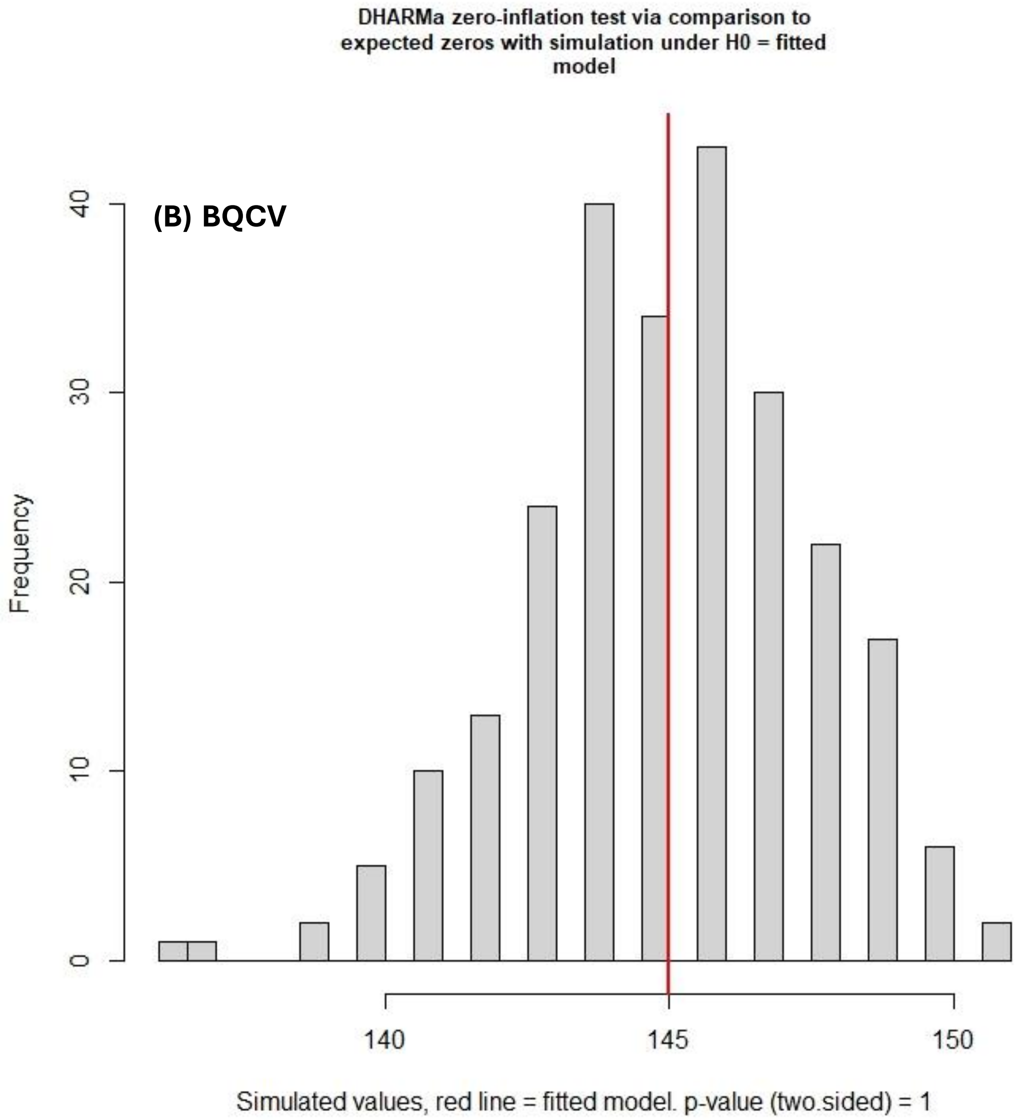
DHARMa zero-inflation test via comparison to expected zeros with simulation under H0 = fitted model
(B) BQCV
Frequency
0
10
20
30
40
140
145
150
Simulated values, red line = fitted model. p-value (two.sided) = 1

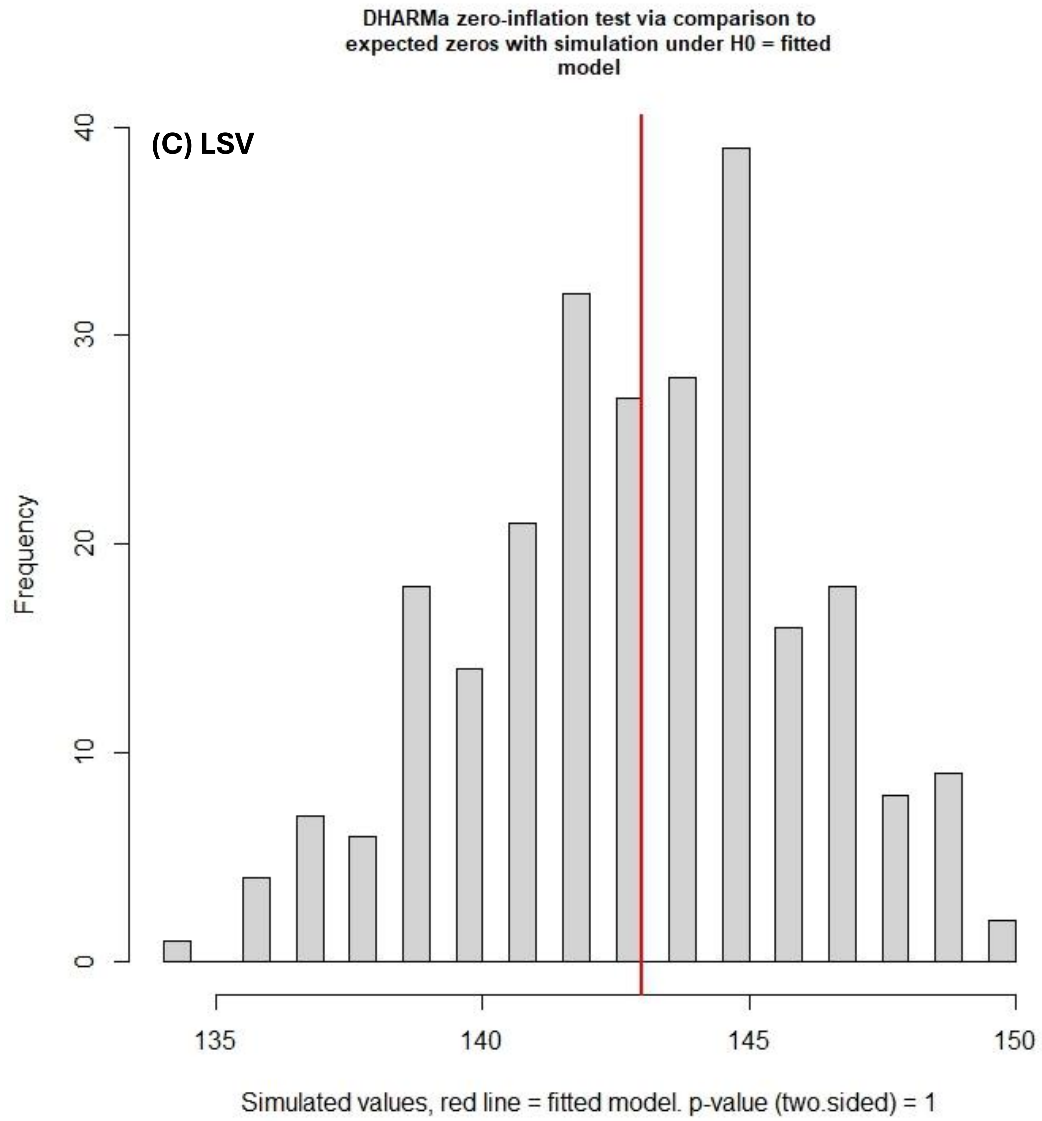
DHARMa zero-inflation test via comparison to expected zeros with simulation under H0 = fitted model
(C) LSV
Frequency
0
10
20
30
40
135
140
145
150
Simulated values, red line = fitted model. p-value (two.sided) = 1

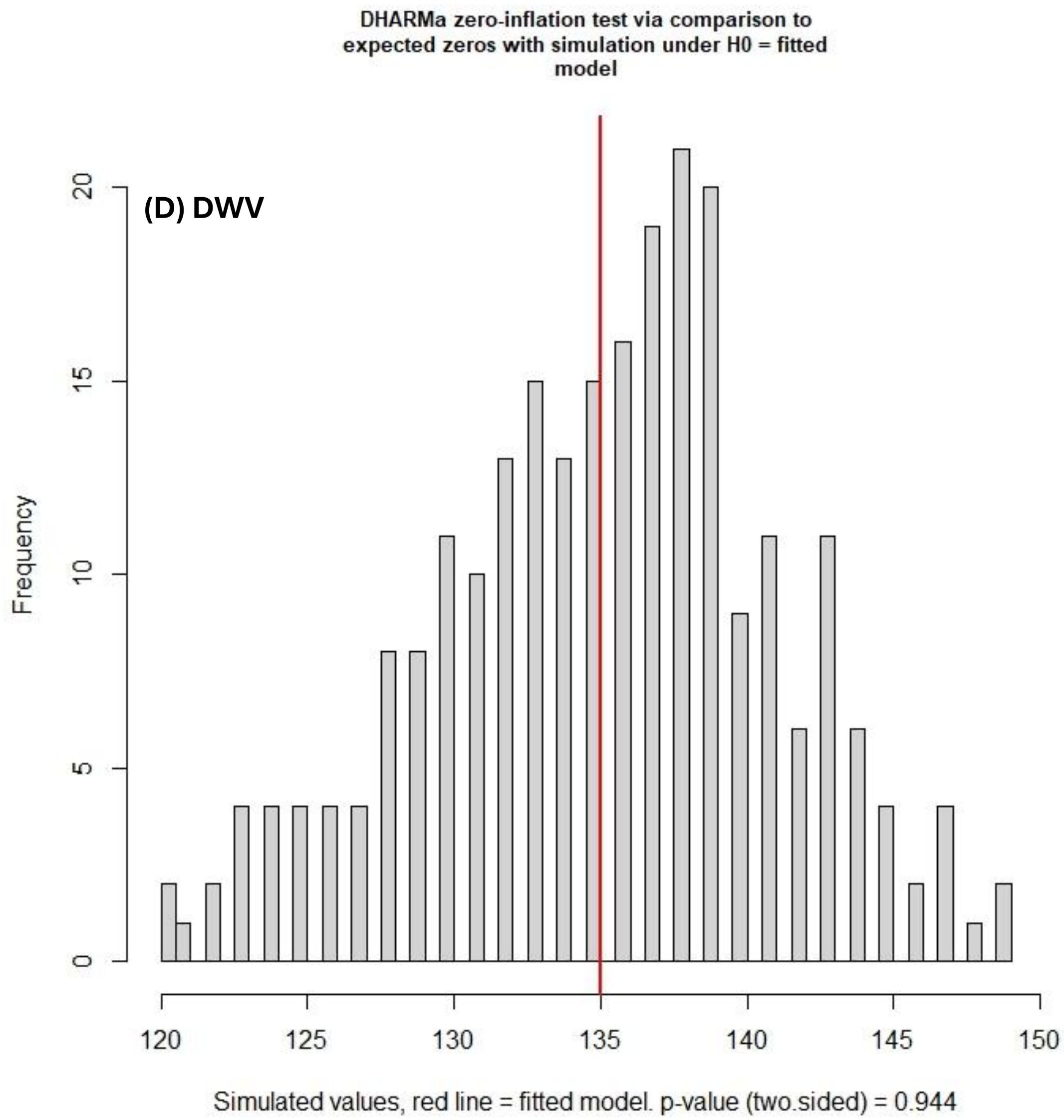

Figure S1 — DHARMa zero inflation diagnostic plots for each virus model. These plots visually confirm the results of the formal tests, showing that observed zero counts fall within the expected simulation envelopes.

References

Brooks, M. E., K. Kristensen, K. J. Van Benthem, A. Magnusson, C. W. Berg, A. Nielsen, H. J. Skaug, M. Mächler and B. M. Bolker. 2017. "glmmTMB balances speed and flexibility among packages for zero-inflated generalized linear mixed modeling." *The R Journal* **9**(2): 378–400.

Hartig, F. and L. Lohse. 2022. "DHARMa: Residual diagnostics for hierarchical (Multi-level/mixed) regression models." Version 0.4.6. https://cran.r-project.org/package=DHARMa.
