# Supplementary material for: Virus distributions in wild bees are associated with floral communities at local to landscape scales
Source: Ecol Appl. 2025 Nov 11;35(7):e70133. doi: 10.1002/eap.70133 (PMC12604080; doi:10.1002/eap.70133)
Supplement: Supplementary file 3 — Appendix S3. [file EAP-35-e70133-s004.pdf]

Virus distributions in wild bees are associated with floral communities at local to landscape scales

Idan Kahnonitch, Katie F. Daughenbaugh, Na'ama Arkin, Tal Erez, Achik Dorchin, Michelle L. Flenniken, Nor Chejanovsky, Asaf Sadeh, Yael Mandelik

Ecological Applications

Appendix S3

Table S1. Flower species scores on the first two CA ordination axes (list A. CA1 axis, list B. CA2 axis), computed using the function "scores", with no transformations (vegan package; Oksanen et al., 2020). As CA1 axis is negatively correlated with LSV-2 in mining bees, flower taxa score values on **CA1 (A)** indicate the direction and strength of their association with LSV-2 prevalence in mining bees. As CA2 axis correlated positively with BQCV in mining bees, flower taxa score values on **CA2 (B)** indicate the direction and strength of its association with BQCV prevalence in mining bees. CA1 and CA2 axes represent 31.95% and 22.61% respectively of the total explained variance in floral composition among the study sites.

(A)

**Flower taxa**	**Score on CA1**
Catananche lutea	8.40
Trifolium spp.	7.15
Notobasis syriaca	3.36

Anthemis spp.	2.95
Calendula spp.	2.72
Salvia dominica	2.72
Carduus argentatus	2.63
Echium judaeum	2.55
Pallenis spinosa	1.58
Ornithogalum narbonense	0.70
Ferula communis	0.65
Chaetosciadium trichospermum	-0.02
Silybum marianum	-0.08
Lathyrus hierosolymitanus	-0.14
Glebionis coronarium	-0.15
Lotus spp.	-0.17
Yellow Brassicaceae *	-0.18
Prasium majus	-0.26
Malva spp.	-0.28
Vicia villosa	-0.41
Erucaria hispanica	-0.51
Isatis lusitanica	-0.58
Ononis pubescens	-0.58

**Sinapis alba*, *Hirschfeldia incana*, and *Rapistrum rugosum*

(B)

**Flower taxa**	**Score on CA2**

Catananche lutea	4.72
Trifolium spp.	2.67
Carduus argentatus	1.61
Pallenis spinose	1.56
Anthemis spp.	1.37
Ornithogalum narbonense	0.91
Isatis lusitanica	0.57
Ononis pubescens	0.57
Erucaria hispanica	0.43
Vicia villosa	0.26
Yellow Brassicaceae *	0.08
Malva spp.	0.04
Chaetosciadium trichospermum	0.02
Lathyrus hierosolymitanus	-0.04
Prasium majus	-0.33
Lotus spp.	-0.39
Glebionis.coronarium	-0.43
Silybum marianum	-0.68
Ferula communis	-2.29
Notobasis syriaca	-3.46
Echium judaeum	-6.96
Calendula spp.	-7.47
Salvia dominica	-7.47

**Sinapis alba*, *Hirschfeldia incana*, and *Rapistrum rugosum*

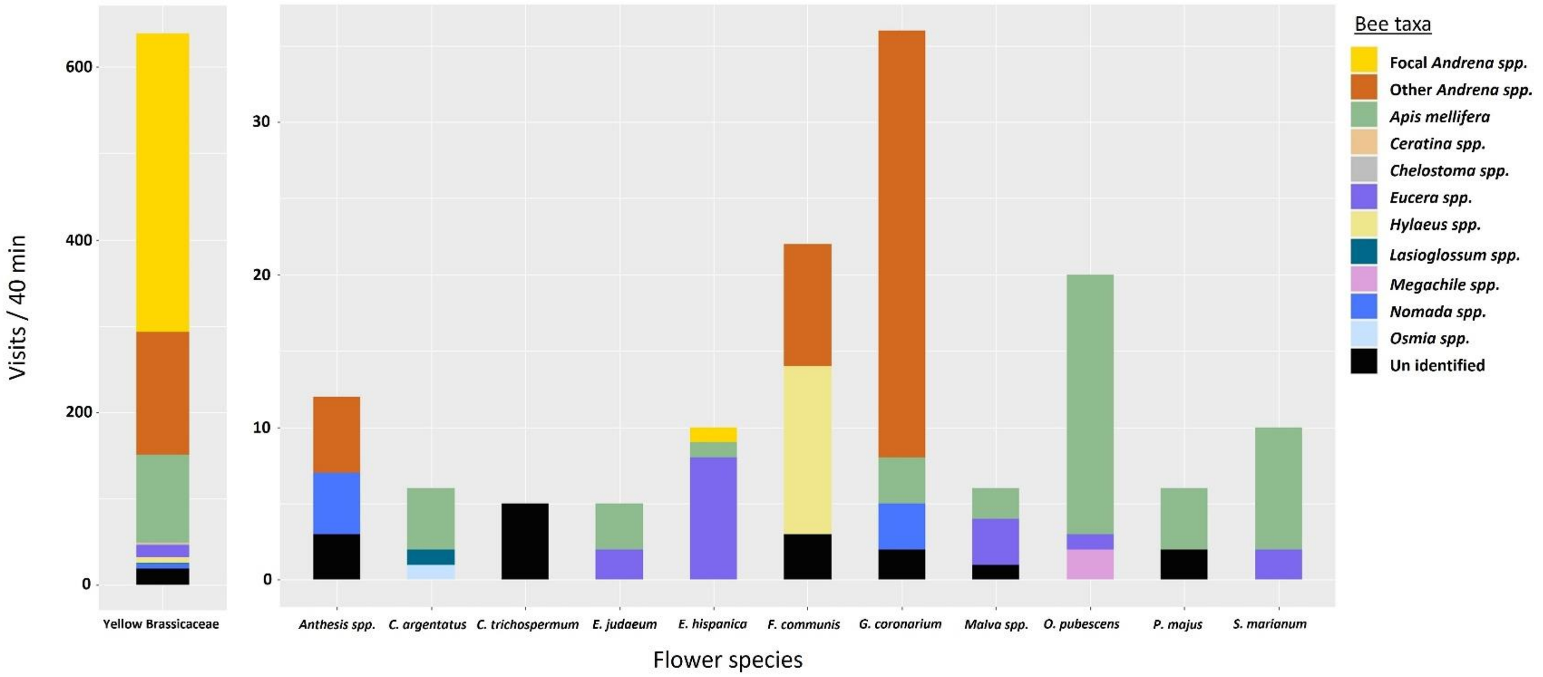

Figure S1. The distribution of floral visits among the bees recorded in field survey. Presented are floral plant taxa (X axis) which together received 97.6% of overall recorded visits (upper median). Yellow Brassicaceae include the compiled visits recoded on *Sinapis alba, Hirschfeldia incana,* and *Rapistrum rugosum*. Focal *andrena spp.* include the three most dominant *Andrena* species: *A. ocraceohirta*, *A. aerinifrons levantina*, *A. urfanella*, and a closely related morphospecies from the subgenus *Truncandrena*. Note the scale difference in the number of visits recorded on the yellow Brassicaceae and the rest of the floral species in the plot.

References

Oksanen, J., F. G. Blanchet, R. Kindt, P. Legendre, P. R. Minchin, R. B. O'Hara, G. L. Simpson, P. Solymos, M. H. H. Stevens, and H. Wagner. 2020. "vegan: Community Ecology Package." R Package Version 2.5-6. https://CRAN.R-project.org/package=vegan.
