# Supplementary material for: Virus distributions in wild bees are associated with floral communities at local to landscape scales
Source: Ecol Appl. 2025 Nov 11;35(7):e70133. doi: 10.1002/eap.70133 (PMC12604080; doi:10.1002/eap.70133)
Supplement: Supplementary file 4 — Appendix S4. [file EAP-35-e70133-s003.pdf]

Virus distributions in wild bees are associated with floral communities at local to landscape scales

Idan Kahnonitch, Katie F. Daughenbaugh, Na'ama Arkin, Tal Erez, Achik Dorchin, Michelle L. Flenniken, Nor Chejanovsky, Asaf Sadeh, Yael Mandelik

Ecological Applications

Appendix S4

The Floral Resource Availability (FRA) index represents a relatively new approach in pollinator ecology research, differing from the traditional method of using the proportion of specific land-cover types (e.g. the proportion of non-agricultural land) for landscape evaluation. It is based on assigning each land-cover type an expert-based relative value that reflects its typical floral resource density during the relevant blooming period. In our study, FRA was calculated as the weighted average value of the different land cover types around each site. Based on AIC, using the proportion of non-agricultural land as the landscape-level predictor, provided models equivalent to the FRA models only in the analysis of SBV prevalence (Table S1); we therefore discuss the models with the proportion of non-agricultural land only for this specific case. Six equivalently fitted models were selected for SBV prevalence in mining bees, consisting of the following predictors: (1) the proportion of non-agricultural land, with negative association within 500 or 250 m from the site, in five of the models, and with marginally positive association within 1000 m from the site, in one model, (2) the estimated density of SBV-

positive *A. mellifera* foragers, with positive association, in three of the models, and (3) floral composition (1st CA axis) in the other three models, together with (4) floral diversity (positively associated with SBV in mining bees) in one model (Table S2). The negative association found between the proportion of non-agricultural land and SBV prevalence in mining bees may be attributed to increased exposure to pesticides (Krupke 2012), a factor linked to increased SBV prevalence in wild bees sampled from agricultural landscapes (Tsvetkov et al., 2021).

Table S1: Difference in AICc between start analysis (% non-agricultural) and second analysis (FRA) of top models predicting virus prevalence in *Andrena*.

Virus	ΔAICc (start to second analysis)
BQCV	2.32
DWV	Spatial variable was not included in the top models
LSV-2	3.53
SBV	0.02

Table S2: Selected models (within 2 AICc units of top model and >2 AICc units from the null model) of virus presence in ***Andrena***, using the proportion of non-agricultural land as the landscape-scale predictor

Selected models (within 2 AICc units of top model and >2 AICc units from the null model) of virus presence in ***Andrena***, using the proportion of non-agricultural land as the landscape-scale predictor. For each predictor included in a model, we present the sign and magnitude of its scaled coefficient, and statistical significance at P<0.01 (**), P<0.05 (*), or P<0.1 (·). Statistically non-significant predictors included in the models are indicated as n.s., and excluded predictors are indicated with dashes. We also indicate in parentheses the best-fitting range of the landscape predictor, and correspondence analysis (CA) axis, and the measure of honey bee density (total or infected only). Gray shading indicates that the null model was either the best or equivalent to the best model.

	Local floral community		Proportion of Non-agricultural land	*A. mellifera density*
	Flower species diversity	Flower genus composition		
**BQCV**	————			
**DWV**				(total)
Model 1	**8.65****	----	----	**3.25****
**LSV-2**	————			
**SBV**		(CA1)		(infected)
Model 1	----	**-1.90****	**-119.35**** (500 m range)	----
Model 2	----	----	**-27.43*** (500 m range)	**4.60****
Model 3	----	----	**8.95·** (1000 m range)	**7.55****
Model 4	----	----	**-24.15·** (250 m range)	**4.86****

Model 5	n.s.	**-2.23****	**-91.17**** (500 m range)	----
Model 6	**7.82****	**-2.81****	**-54.04**** (250 m range)	----

- For each predictor included in a model we present the sign and magnitude of its scaled coefficient, and statistical significance at P<0.01 (**), P<0.05 (*), or P<0.1 (·).
- Statistically non-significant predictors included in the models are indicated as n.s., and excluded predictors are indicated with dashes.
- We indicate in parentheses the best-fitting range of the landscape predictor, and correspondence analysis (CA) axis, and the measure of honey bee density (total or infected only).
- Long dash indicates that the null model was either the best or equivalent to the best model; coefficients not presented.

References

Krupke, C. H., G. J. Hunt, B. D. Eitzer, G. Andino, and K. Given. 2012. “Multiple routes of pesticide exposure for honey bees living near agricultural fields.” *PLoS one* **7**(1): e29268.

Tsvetkov, N., V. J. MacPhail, S. R. Colla, and A. Zayed. 2021. "Conservation genomics reveals pesticide and pathogen exposure in the declining bumble bee *Bombus terricola*." Molecular ecology **30**(17): 4220-4230.
