# Supplementary material for: Virus distributions in wild bees are associated with floral communities at local to landscape scales
Source: Ecol Appl. 2025 Nov 11;35(7):e70133. doi: 10.1002/eap.70133 (PMC12604080; doi:10.1002/eap.70133)
Supplement: Supplementary file 5 — Appendix S5. [file EAP-35-e70133-s001.pdf]

Virus distributions in wild bees are associated with floral communities at local to landscape scales

Idan Kahnonitch, Katie F. Daughenbaugh, Na'ama Arkin, Tal Erez, Achik Dorchin, Michelle L. Flenniken, Nor Chejanovsky, Asaf Sadeh, Yael Mandelik

Ecological Applications

Appendix S5

Table S1: Detailed statistical results for the best models for the roles of *A. mellifera* and flower plant community on the probability of virus infection (SBV, DWV, BQCV, LSV-2) in *Andrena* bees, using the **FRA index** as the landscape-scale variable. All models are binomial GLMMs for the probability of virus presence in *Andrena*. Site was incorporated as a random intercept factor. All reduced-term model variations yielded ΔAICc values > 2. The best model was contrasted with a null model containing only the random effect of site.

**Explanatory variables**	**Effect**	**SE**	**LRT**	***P***
BQCV				
Model 1	ΔAICc (from null model) = 2.32			
Landscape flower score (1000m radius)	28.10	12.30	4.86	0.0276
Floral composition (CA2)	2.02	1.00	3.87	0.0491

Model 2	ΔAICc (from best model) = 0.26			
Landscape flower score (1000m radius)	40.39	17.06	6.66	0.0099
Floral composition (CA2)	2.20	1.14	4.31	0.0380
A. mellifera forager density	-0.71	0.51	1.88	0.1700
DWV				
Model 1	ΔAICc (from null model) = 7.71			
A. mellifera forager density	3.25	1.54	8.98	0.0028
Floral diversity	8.65	4.72	11.45	0.0007
LSV-2				
Model 1	ΔAICc (from null model) = 4.64			
Landscape flower score (250m radius)	13.86	7.98	6.97	0.0083
Floral composition (CA1)	-3.85	2.53	7.50	0.0062
Model 2	ΔAICc (from best model) = 1.19			
Landscape flower score (250m radius)	15.57	8.25	7.91	0.0049
Floral composition (CA1)	-3.93	2.64	6.15	0.0132
Floral diversity	-1.13	1.15	0.94	0.3312

<u>SBV</u>				
Model 1	ΔAICc (from null model) = 9.66			
Infected *A. mellifera* forager density	7.80	2.63	12.52	0.0004
Landscape flower score (250m radius)	10.60	7.55	4.71	0.0300
Model 2	ΔAICc (from null model) = 0.45			
Infected *A. mellifera* forager density	8.10	2.67	12.35	0.0004
Landscape flower score (500m radius)	8.73	5.88	4.25	0.0392

Table S2: Detailed statistical results for the best models for the roles of *A. mellifera* and flower plant community on the probability of virus infection (SBV, DWV, BQCV, LSV-2) in *Andrena* bees, using the **proportion of non-agricultural** land as the landscape-scale variable. All models are binomial GLMMs for the probability of virus presence in *Andrena*. Site was incorporated as a random intercept factor. All reduced-term model variations yielded ΔAICc values > 2. The best model was contrasted with a null model containing only the random effect of site.

**Explanatory variables**	**Effect**	**SE**	**LRT**	***P***
<u>BQCV</u>				
Null model (site + intercept only)	ΔAICc (from null model) = 0			

<u>DWV</u>				
Best model	ΔAICc (from null model) = 7.71			
A. mellifera forager density	3.25	1.54	8.98	0.0028
Floral diversity	8.65	4.72	11.45	0.0007
<u>LSV-2</u>				
Model 1	ΔAICc (from null model) = 1.11			
<u>SBV</u>				
Model 1	ΔAICc (from null model) = 9.64			
Floral composition (CA1)	-1.90	0.67	7.49	0.0062
% of non-cultivated land (500m radius)	-119.35	41.17	13.82	0.0002
Model 2	ΔAICc (from top model) = 0.606			
Infected *A. mellifera* forager density	4.60	1.46	6.89	0.0087
% of non-cultivated land (500m radius)	-27.43	29.10	4.08	0.0433
Model 3	ΔAICc (from top model) = 0.91			
Infected *A. mellifera* forager density	7.55	2.38	11.76	0.0006

% of non-cultivated land (1000m radius)	8.95	6.66	3.78	0.0519
Model 4	ΔAICc (from best model) = 1.28			
Infected *A. mellifera* forager density	4.86	1.47	8.40	0.0038
% of non-cultivated land (250m radius)	-24.15	42.60	-0.57	0.0650
Model 5	ΔAICc (from best model) = 1.66			
Floral composition (CA1)	-2.23	0.83	6.11	0.0134
Floral diversity	2.31	2.95	0.48	0.4885
% of non-cultivated land (500m radius)	-91.17	40.13	13.57	0.0002
Model 6	ΔAICc (from best model) = 1.68			
Floral composition (CA1)	-2.81	0.94	10.09	0.0015
Floral diversity	7.82	3.24	8.59	0.0034
% of non-cultivated land (250m radius)	-54.04	31.08	13.55	0.0002
